# Integrating single‐cell RNA sequencing with spatial transcriptomics reveals an immune landscape of human myometrium during labour

**DOI:** 10.1002/ctm2.1234

**Published:** 2023-04-24

**Authors:** Kaiyuan Ji, Lina Chen, Xiaodi Wang, Bolun Wen, Fan Yang, Wenfeng Deng, Yunshan Chen, Guozheng Zhang, Huishu Liu

**Affiliations:** ^1^ Guangzhou Key Laboratory of Maternal‐Fetal Medicine Guangzhou Women and Children's Medical Center, Guangzhou Medical University Guangzhou China; ^2^ School of Medicine South China University of Technology Guangzhou China

**Keywords:** inflammation, labour, myometrium, single‐cell RNA‐seq, spatial transcriptomics

## Abstract

**Background:**

The transition of the myometrium from a quiescent to a contractile state during labour is known to involve inflammation, which is characterized by the infiltration of immune cells and the secretion of cytokines. However, the specific cellular mechanisms underlying inflammation in the myometrium during human parturition are not yet fully understood.

**Methods:**

Through the analysis of transcriptomics, proteomics, and cytokine arrays, the inflammation in the human myometrium during labour was revealed. By performing single‐cell RNA sequencing (scRNA‐seq) and spatiotemporal transcriptomic (ST) analyses on human myometrium in term in labour (TIL) and term in non‐labour (TNL), we established a comprehensive landscape of immune cells, their transcriptional characteristics, distribution, function and intercellular communications during labour. Histological staining, flow cytometry, and western blotting were applied to validate some results from scRNA‐seq and ST.

**Results:**

Our analysis identified immune cell types, including monocytes, neutrophils, T cells, natural killer (NK) cells and B cells, present in the myometrium. TIL myometrium had a higher proportion of monocytes and neutrophils than TNL myometrium. Furthermore, the scRNA‐seq analysis showed an increase in M1 macrophages in TIL myometrium. CXCL8 expression was mainly observed in neutrophils and increased in TIL myometrium. CCL3 and CCL4 were principally expressed in M2 macrophages and neutrophils‐6, and decreased during labour; XCL1 and XCL2 were specifically expressed in NK cells, and decreased during labour. Analysis of cytokine receptor expression revealed an increase in IL1R2, which primarily expressed in neutrophils. Finally, we visualized the spatial proximity of representative cytokines, contraction‐associated genes, and corresponding receptors in ST to demonstrate their location within the myometrium.

**Conclusions:**

Our analysis comprehensively revealed changes in immune cells, cytokines, and cytokine receptors during labour. It provided a valuable resource to detect and characterize inflammatory changes, yielding insights into the immune mechanisms underlying labour.

## Graphical abstract

1. An investigation was carried out at the single‐cell level to analyze the alterations in immune cells and cytokines in both terms in labour and non‐labour myometrium.

2. Term in labour myometrium had a higher proportion of monocytes and neutrophils than non‐labour myometrium, especially M1 macrophages.

3. Myometrial inflammation was enhanced during labour and related to the strengthen of uterine contraction.

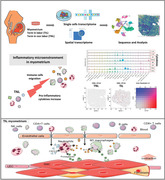

## 1 INTRODUCTION

The uterus is a myogenic organ, primarily responsible for nurturing and protecting the fetus and giving birth after prolonged contractile activity. Term labour has been considered an inflammatory event, which is closely related to combination of adaptive and humoral immune responses and mechanical factors.^1^, ^2^, ^3^ Circulating maternal monocytes and leukocytes are recruited at the maternal/fetal interface and other reproductive tissues.^4^, ^5^ Myometrium is the largest component of uterus and contracts intensely during labour, which is indispensable for successful delivery.^5^, ^6^ Mounting evidence has shown the importance of inflammation in the myometrium for spontaneous term labour.^7^ However, the inflammatory response in the myometrium during labour is a complicated and currently unclear process. Thanks to the advent of high‐throughput sequence and high‐resolution molecular detection techniques, it is possible to study the inflammatory response in the myometrium at the single cell level.

Studies have shown an association between myometrial inflammation and term labour.^3^, ^8^ The inflammatory process generally involves immune cell infiltration and cytokines production. In fact, studies have demonstrated the presence of proinflammatory immune cells and cytokines in reproductive tissues and at the maternal‐fetal interface during term or preterm labour.^9^, ^10^, ^11^ Several studies have demonstrated increased leukocyte infiltration in the myometrium during labour. In fact, a significant increase of the proportion of neutrophils and macrophages was observed in the lower segment of the myometrium.^12^, ^13^ Leukocytes are the main sources of the increased myometrial inflammatory cytokines such as interleukin‐1 (IL‐1), IL‐6 (IL6), IL‐8 (CXCL8), and tumor necrosis factor (TNF)‐alpha,^14^, ^15^ which are responsible for increasing the contractility of myometrial cells.^14^, ^16^, ^17^, ^18^, ^19^, ^20^, ^21^, ^22^ A study has shown a small number of mast cells in the myometrium during late gestation,^23^ which modulates uterine contractility via degranulation or the effects of their mediators.^24^ Additionally, the activation of NK cells, T cells, and dendritic cell (DC) in the myometrium play a role during preterm labour.^25^ Further, application of microarray and RNA‐sequencing to study the transcriptomic of the labouring and non‐labouring myometrium, and verified that overexpression of pro‐inflammatory cytokines and chemokines during labour. Biological process and pathway analyses performed on the differential expressed genes revealed that myometrial inflammation is a key driver of labour.^26^, ^27^, ^28^, ^29^, ^30^ Pro‐inflammatory cytokines, such as IL‐8, IL‐1β, and TNF‐α, stimulate the production of PGE2, which promotes uterine contractions.^31^ Our previous researches on labouring myometrium have also confirmed the infiltration of macrophages and up‐regulation of pro‐inflammatory cytokines like IL‐1β, TNF‐α, IL‐6, IL‐8 and chemokines like MIP‐1β (CCL4).^11^ An integrated transcriptomic and proteomic analysis performed on human labouring myometrium revealed that inflammation response was one of the most enriched biological processes during labour.^32^ Previous studies above have demonstrated a strong correlation between myometrial inflammation and labour. However, these studies had technological limitations to investigate the involvement and interaction of multiple cell types in the myometrium, Studies of cell‐cell interaction, regulation of a specific cell population, and spatial pattern of immune cells in the myometrium require higher resolution techniques

Single‐cell RNA sequencing (scRNA‐seq) is applied for transcriptome capture of individual cells from hybrid tissues, which allows the characterization of the genetic and functional heterogeneities of cells.^33^ A recent study performed scRNA‐seq using human myometrium during term labour, and unraveled the major cell populations including smooth muscle cells (SMC), monocytic cells, fibroblasts, and endothelial cells, with specific transcriptome activity associated with labour‐related inflammatory and contractile processes.^34^ Spatial transcriptome (ST) provides information on the spatial localization of cell types, gene expression patterns, and their proximity to each other, making it a perfect complement to scRNA‐seq.^35^ Therefore, a comprehensive strategy to establish scRNA‐seq and ST analyses will deepen the exploration of labour‐related myometrial inflammation in specific myometrial cell types, which is yet to be developed.

In this study, for the first time, an integrating scRNA‐seq and ST analysis were performed on spontaneous term labouring and non‐labouring myometrium. We focused on the alterations of specific immune cells, cytokines, and cytokine receptors, and revealed their spatial location and interaction, which allows us to trace the source of labour‐related cytokines in the myometrium. Together, this will enable us to generate high‐resolution maps of immune cell and cytokines in myometrium during labour.

## 2 RESULTS

### 2.1 Inflammatory response in myometrium during labour

**FIGURE 1:**
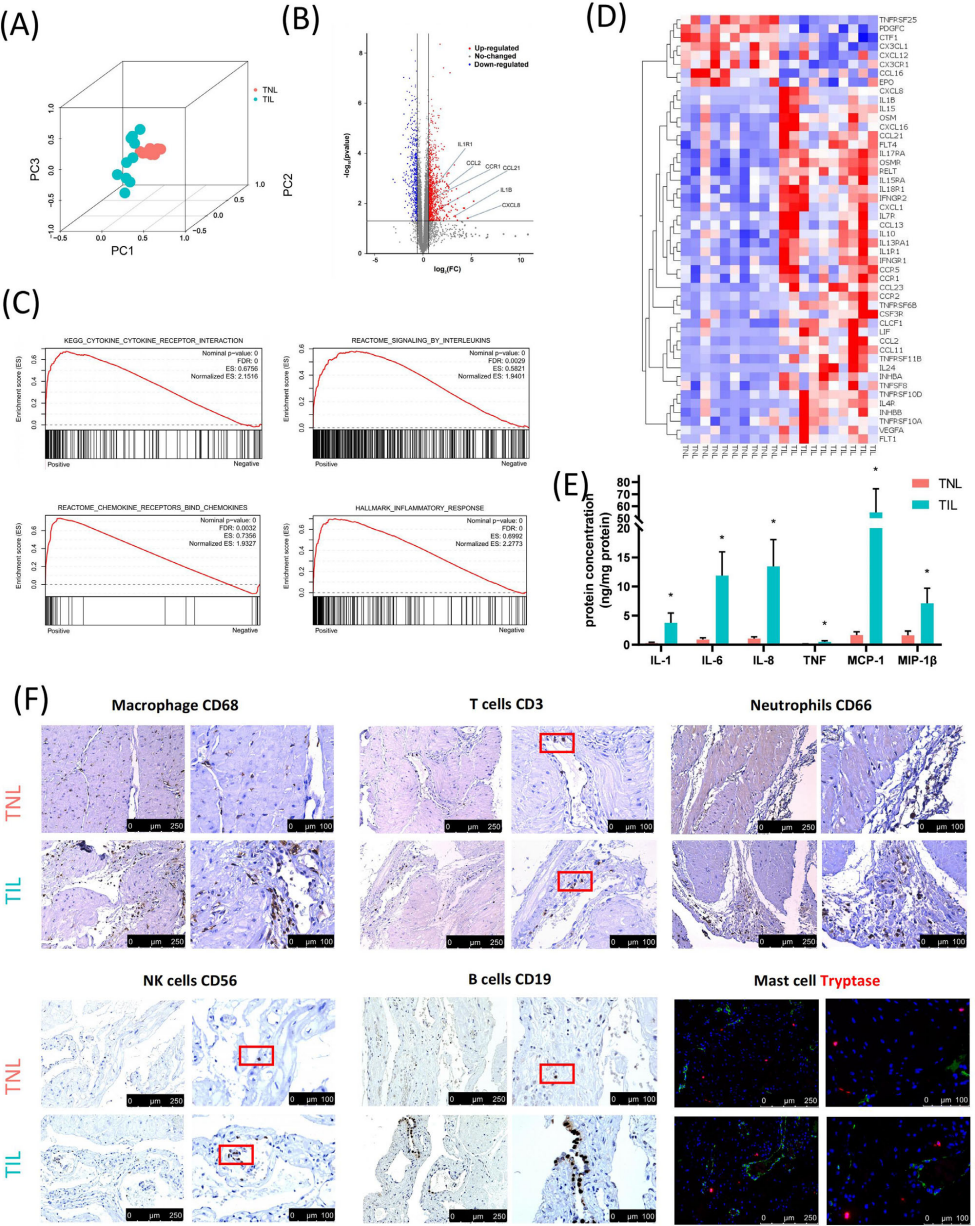
Overview of cytokines and immune cells in human myometrium during labour. (A, B) principal component analysis (PCA) and volcano plot of the changes in the transcriptome (bulk RNA‐seq, GSE137552) in the myometrium from the term in non‐labour (TNL) (*n* = 10) and term in labour (TIL) (*n* = 10) groups (C) Gene Set Enrichment Analysis for bulk RNA‐seq of the myometrium from the TNL (*n* = 10) and TIL (*n* = 10) groups (GSE137552) indicated the increase of cytokines and inflammatory responses during labour. (D) Heatmap of the DEGs (adj *p*‐value < 0.05 and |fold change| > 1.5) of cytokines and receptors from KEGG cytokines list (map04060) in bulk RNA‐seq (GSE137552). (E) Quantification of cytokines by Luminex assays in the myometrium from the TNL (*n* = 10) and TIL (*n* = 10) groups. *p < 0.05. (mean ± standard deviation, *t*‐test, two‐tailed) (F) Representative images of the markers of macrophage (CD68), T cells (CD3), neutrophils (CD66), NK cells (CD56), B cells (CD19), and mast cells (Tryptase, red) in the myometrium of TNL and TIL groups. Endothelial cells were colored with VWF (green) for locating the mast cells. Red box indicated some positive cells in the staining sections.

Labour is an inflammatory process. Our previous studies demonstrated and validated the inflammatory response in myometrium during labour using transcriptomics, proteomics, and cytokine arrays, consistent with other studies.^11^, ^32^ Results showed the gene expression changed a lot during labour, especially the genes related to cytokines and chemokine (Figure 1A–C). We listed the differentially expressed genes (DEGs) of cytokine‐cytokine receptor interaction pathway (KEGG database, map04060) in bulk RNA‐seq (Figure 1D). CXCL8, IL1B, CCL21 and CCL2 were distinctly up‐regulated in TIL myometrium. Cytokines arrays of TNL and TIL myometrium showed IL‐1, IL‐6, IL‐8, MCP‐1, TNF and MIP‐1β increased during labour (Figure 1E). Immunohistochemistry or immunofluorescence were performed on human myometrium in TIL and TNL to study the localization of immune cell like macrophages, T cells, neutrophils, NK cells, B cells, and mast cells (Figure 1F and S1A). All these immune cells could be found in the myometrium in TIL and TNL. Macrophages, T cells and neutrophils were more numerous than that of other immune cells. The changes observed in the immune cell and cytokine levels within the myometrium appeared to be a highly coordinated process, likely contributing to the enhanced uterine contractions during labour.

### 2.2 scRNA‐seq and ST analysis of the TNL and TIL myometrium

**FIGURE 2:**
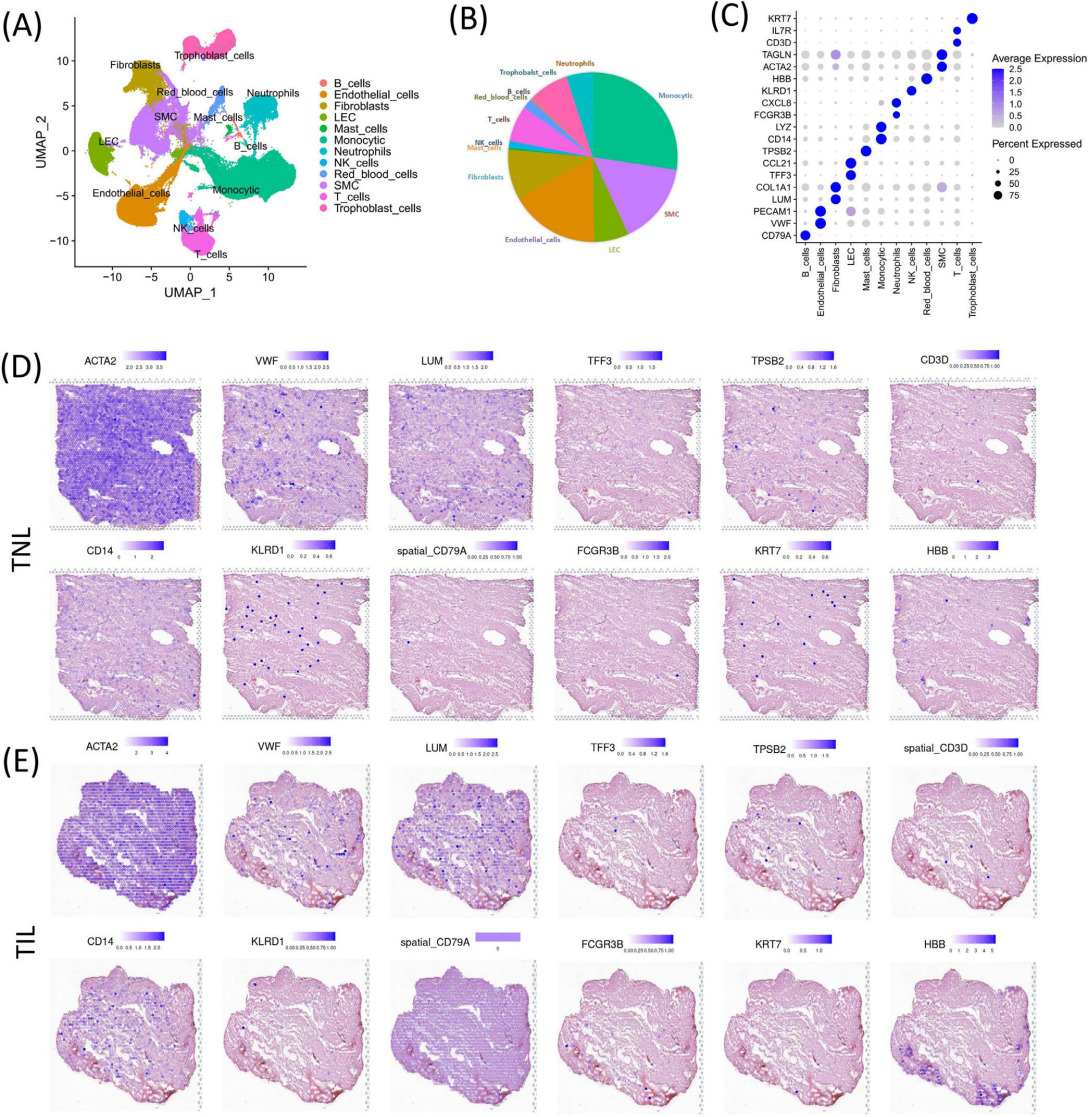
Overall landscapes of the single‐cell RNA‐sequence (scRNA‐seq) and spatial transcriptomics (ST) in the myometrium of the term in non‐labour (TNL) and term in labour (TIL) groups. (A) scRNA‐seq analysis displays the UMAP map of the major myometrial cell populations from TNL (*n* = 5) and TIL (*n* = 5) groups. (B) Proportion of each cell population in scRNA‐seq data. (C) Dot plot of the expression of canonical markers for each cell population in scRNA‐seq data. (D, E) Spatial distribution of canonical markers for each cell population were displayed using ST data of the myometrium of TNL (*n* = 1) and TIL (*n* = 1) groups.

To provide a comprehensive atlas of the inflammatory response during labour in the myometrium, scRNA‐seq and ST analysis was performed on the myometrium collected from women in TIL (scRNA‐seq: *n* = 5; ST: *n* = 1) and TNL (scRNA‐seq: *n* = 5; ST: *n* = 1) (Table S1). After the standard quality control analysis of scRNA‐seq data (Figure S1B), we obtained single‐cell transcriptomes from a total of 121,834 single cells, of which 63,460 cells were from myometrium in the TNL group, and 58,374 cells were from myometrium from the TIL group. To define cell types in scRNA‐seq, the Seurat (version 4.0.2) was used for normalization, batch effect correction, and principal component analysis (PCA). Cell clustering was performed by unsupervised uniform manifold approximation and projection (UMAP), a total of 12 cell populations were identified: (1) B cells (CD79A+), (2) endothelial cells (VWF+), (3) fibroblasts (LUM+), (4) lymphoid endothelial cells (LEC) (TFF3+), (5) mast cells (TPSB2+), (6) monocytic cells (CD14+), (7) neutrophils (FCGR3B+), (8) NK cells (KLRD1+), (9) red blood cells (HBB+), (10) SMCs (ACTA2+), (11) T cells (CD3D+), and (12) trophoblast cells (KRT7+) (Figure 2A and Figure S1D). The cell types identity were almost consistent with previous studies and canonical markers.^34^, ^36^, ^37^, ^38^ Cell number and proportion of each cell population listed in Figure 2B and Table S2. Trophoblast cells were also detected in the myometrial tissues (Figure S7A). Previous studies have reported that these trophoblast cells could be scattered placental cells attached to the myometrial tissues.^34^ Hence, we did not further analyze the trophoblast cells in this study until they are verified more in future. The differential expression of the cell type‐specific marker genes was analyzed to match the biological annotation (Figure 2C, Figure S1E and Table S3).

A total of 4285 specific captured areas (spots) in myometrium in the TNL group and 1850 spots in myometrium in the TIL group were identified by ST analysis (Figures S1C and S2A). The canonical markers of each cell population were displayed in the ST arrays (Figure 2D,E). Further, the expression of ACTA2 (SMC marker) was high in most spots in the ST. Whereas less evident spatial characteristics of the markers of B cells, T cells, trophoblast cells, neutrophils and NK cells were found. To predict the cell type of spots, integration of scRNA‐seq and ST was performed and results showed that most spots were mapped to SMC (Figure S2B).

### 2.3 Difference of cell populations and cell type‐specific cytokines in myometrium in the TNL and TIL groups

**FIGURE 3:**
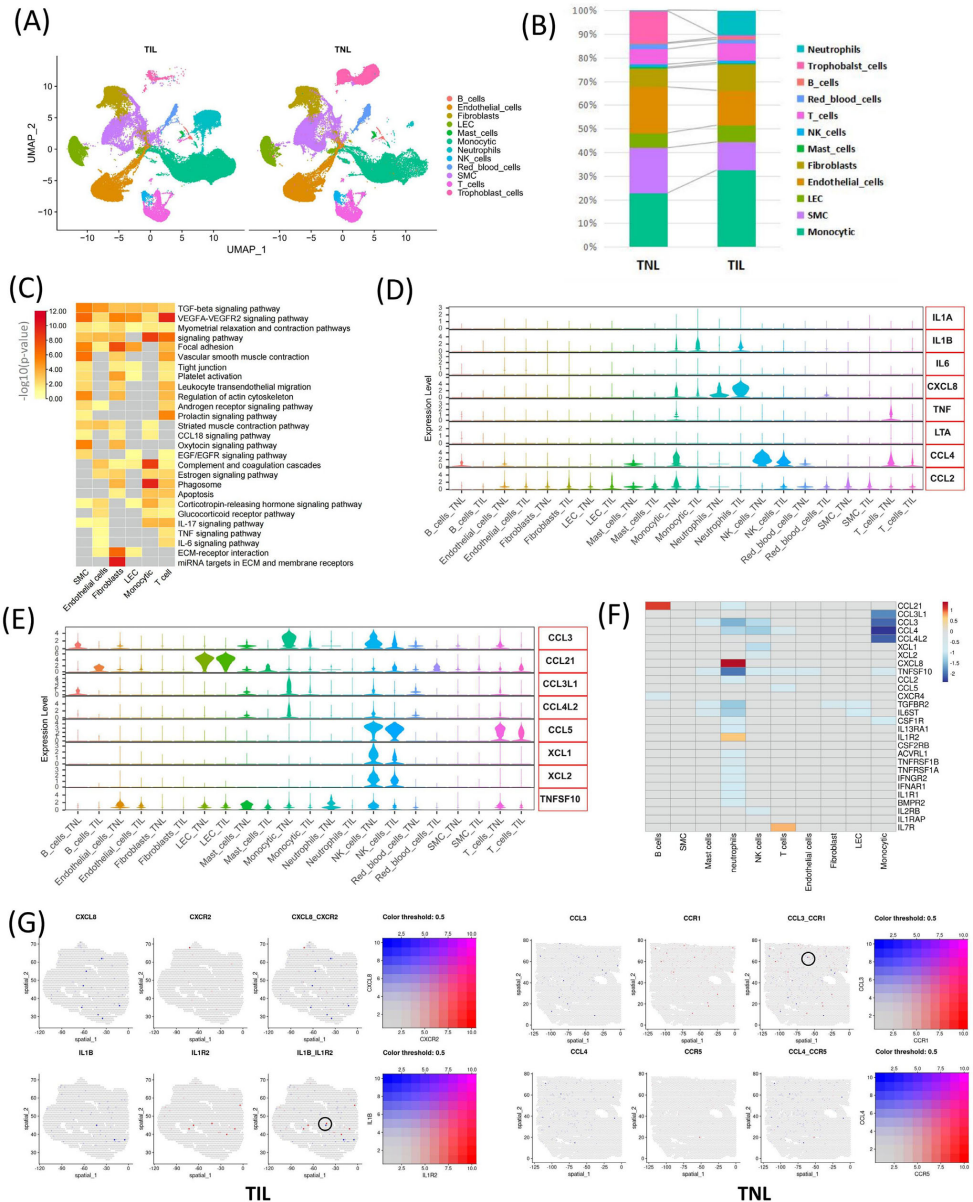
Changes of immune cells and cytokines in various cell types in the myometrium between the term in non‐labour (TNL) and term in labour (TIL) groups. (A) UMAP visualization of the major myometrial cell populations identified using the scRNA‐seq. (B) The proportion of cell subpopulations in the myometrium of TNL and TIL groups which identified using the scRNA‐seq. (C) Heatmap of the representative pathways enriched by DEGs (adj *p*‐value < 0.05 and |fold change| > 1.5) in each cell population. (D) Violin plot of the expression of cytokines, which corresponding to Figure 1E, in each cell population. (E) Violin plots of the expression of other cytokines in each cell population that were differentially expressed (adj *p*‐value < 0.05 and |fold change| > 1.5) in the myometrium between the TNL and TIL groups which were identified using scRNA‐seq. (F) Heatmap shows the differentially expressed cytokines and receptors (adj *p*‐value < 0.05 and |fold change| > 1.5) in each cell population. (G) Spatial distribution of representative differentially expressed cytokines (CXCL8, IL1B, CCL4 and CCL4) and receptors of the myometrium from the TNL and TIL groups. Circles indicate the adjacent expressed cytokines and their receptors.

We next compared the proportion of each cell type in TNL and TIL myometrium (Figures 3A,B). The proportion of neutrophils and monocytes was significant increased in the myometrium of TIL group (Figure 3B). The myometrium undergoes significant biological changes during labour. Hence, to explore the changes in biological processes at the cellular level, we performed pathway enrichment analyses on the differential expressed genes by each main cell type (number with > 1000 cells in both TNL and TIL) (Table S4). Multiple inflammatory‐related pathways were enriched in most cell types, and most cell types were involve in myometrial relaxation and contraction pathways (Figure 3C).

To explore the cytokine array results shown in Figure 1E on a single cell level, the expression (and they are involved) of genes were displayed in each cell population (Figure 3D). The levels of IL1B and CXCL8 were increased in the myometrium of TIL group, consistent with the results of the cytokine array (Figure 3E). IL‐1 (IL1B) was primarily expressed by monocytes and neutrophils. IL‐8 (CXCL8) was expressed by the trophoblast cells, monocyphils. IL1R2 (IL1 receptor) was expressed by neutrophils and trophoblast cells; and the expression of IL1R2 was increased in neutrophils in the myometrium of TIL group. IL1R1 expressed in endothelial cells, fibroblasts, and trophoblast cells, and showed no significant difference between TNL and TIL groups (Figure S3C,D). CXCL8 receptors were expressed in neutrophils (Figure 3D). In addition, IL‐6 (IL6), IL1A, and TNF (TNF and LTA) were expressed in only a few cell types (Figure 3D). TNF receptors were expressed by the monocytes and neutrophils and decreased in TIL neutrophils (Figure S3C,D). IL6ST (IL6 receptor) was decreased in TIL myometrium and only a few cell types expressed IL6R (Figure S3C). MCP‐1 (CCL2) was primarily expressed by mast cells and monocytes; MIP‐1β (CCL4) was expressed by mast cells, NK cells, T cells and monocytes (Figure 3D). The scRNA‐seq results revealed decreased expression of MCP‐1 (CCL2) and MIP‐1β (CCL4) during labour (Figure 3D).

The list of genes encoding the cytokines and receptors was obtained from the KEGG database (map04060). The differential expressed cytokines were screened to explore changes in the inflammatory response during labour (Figure 3E and Figure S1F and S1G). CCL3 was expressed by the B cells, mast cells, monocytic and NK cells, and decreased in TIL group (Figure 3E). CCL21 was expressed by B cells, mast cells, monocytic and LEC cells, and increased in the B cells in TIL myometrium (Figure 3E). CCL3L1 and CCL4L1 were primarily expressed by monocytes, and the expression of CCL3L1 and CCL4L1 was decreased in monocytes in the myometrium of TIL group (Figure 3E). XCL1 and XCL2 were expressed by NK cells, and a decrease in XCL1 and XCL2 expression was observed in NK cells in the myometrium in the TIL group (Figure 3E). TNFSF10 expressed in most cells types, except B cells, fibroblasts and SMC, the expression of TNFSF10 was decreased in the TIL group. The heat map summarized the differentially expressed cytokines and their receptors in the myometrium of TNL and TIL groups (adj *p* < 0.05 and |fold‐change| > 1.5) (Figure 3F).

The overlap among the high‐confidence gene from Stanfield et al, the DEGs from our previous bulk RNA‐seq of the myometrium (GSE137552), and the genes from KEGG cytokines list (map04060) indicates high confidence for CXCL8, CCL2, IL1B, IL7R, IL4R (Figure S3A). Comparing the DEGs in each cell type in the myometrium of TNL and TIL groups from scRNA‐seq and bulk RNA‐seq (GSE137552), neutrophil was the cell type with the largest number of DEGs. (Figure S3B). We analyzed the differential expressed cytokines from scRNA‐seq and bulk RNA‐seq (GSE137552), and found that the expression pattern of CXCL8, CCL21, and IL7R was consistent (Figures 3F and 1D).

In addition, a significant increase in IL1B expression was observed in TIL myometrium, however, the fold change was less than 1.5 (1.34). Considering its crucial biological roles during labour,^39^, ^40^ we highlighted IL1B and its receptors IL1R1 and IL1R2 in ST arrays of TIL myometrium (Figure 3G and Figure S3E). The other representative cytokines and their receptors in ST arrays indicate the spatial proximity between them, which may be the basis for facilitating the cell‐cell communication in the myometrium (Figure 3G and S3E).

### 2.4 Changes in monocytic subpopulation in the myometrium during labour

**FIGURE 4:**
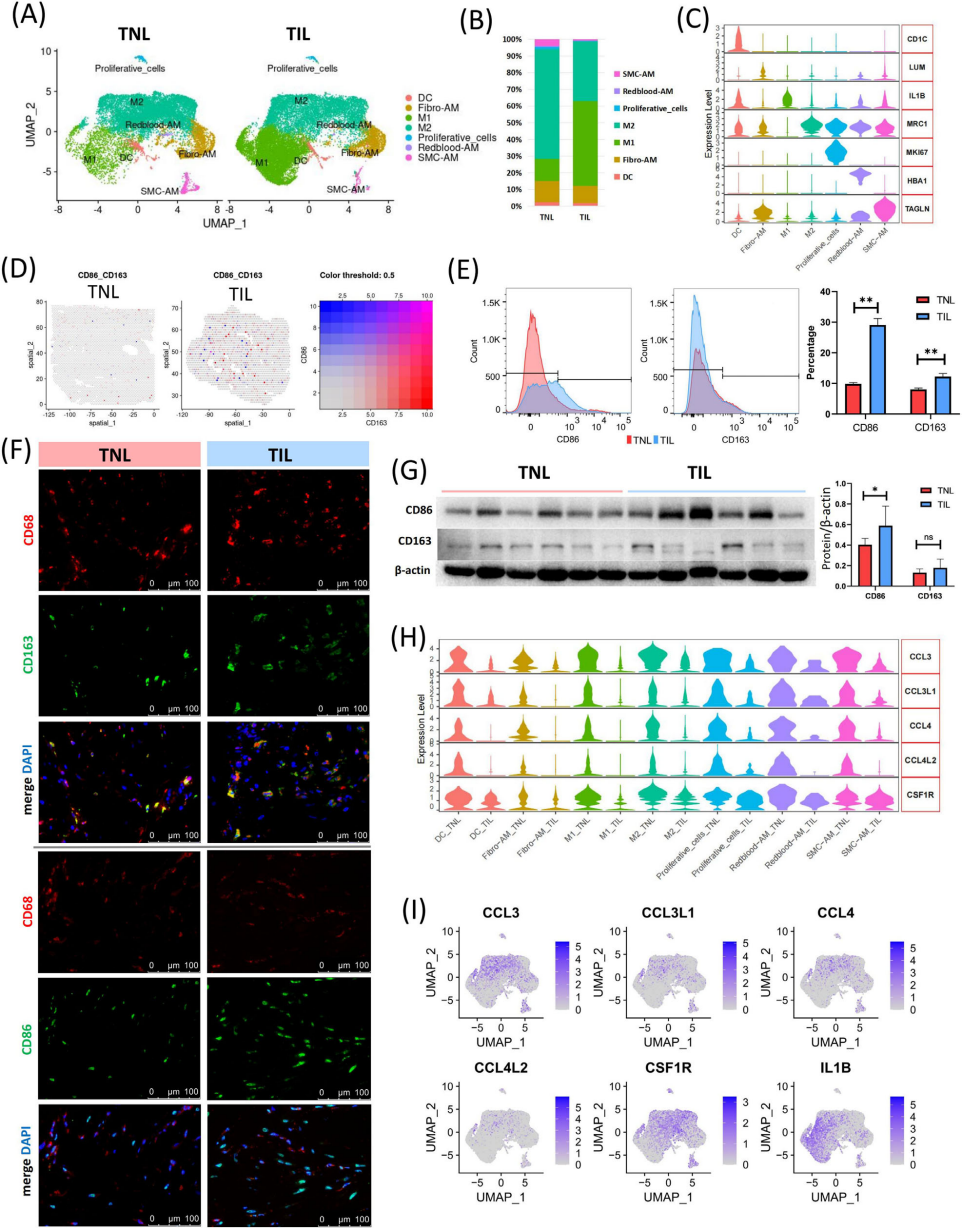
Alteration of monocytes in myometrium during labour. (A) UMAP visualization of monocyte subpopulations divided into groups. DC: dendritic cells; Fibro‐AM: Fibroblast associated macrophages; M1: M1 macrophages; M2: M2 macrophages; Redblood‐AM: Red blood‐associated macrophages; SMC‐AM: SMC‐associated macrophages. (B) The proportion of monocyte subpopulations in the myometrium of the term in non‐labour (TNL) and term in labour (TIL) groups, which was identified using the scRNA‐seq (C) Violin plot of the expression of canonical markers for each monocyte subpopulation in scRNA‐seq. (D) Spatial distribution of M1 (CD86) and M2 (CD163) macrophage markers in the myometrium of TNL and TIL groups. (E) Violin plot of the expression of differentially expressed cytokines and receptors in each monocyte subpopulation. (E) FACS shows the proportion of M1 (CD86) and M2 (CD163) macrophages in the myometrium of TNL and TIL groups (*n* = 3). ** *p* < 0.01. (mean ± standard deviation, t‐test, two‐tailed). (F) Immunofluorescence shows the M1 (CD86) and M2 (CD163) macrophages in the myometrium of TNL and TIL groups. (G) Western blotting (WB) shows the expression of CD86 and CD163 in the myometrium of TNL and TIL groups (*n* = 6). *p < 0.05. (mean ± standard deviation, t‐test, two‐tailed). (H) Violin plot of the expression of differentially expressed cytokines and receptors in each monocyte subpopulation. (I) UMAPs of the expression of differentially expressed cytokines and receptors in monocytes.

Monocytes are the most abundant immune cell types in the myometrium, which play an important part in myometrium during labour. UMAP of scRNA‐seq showed the heterogeneity of monocytes in myometrium. The subpopulation of monocytes identified were DC, macrophages, and proliferative monocytic cells. Of which, macrophages were divided into five subsets: M1 macrophages (M1), M2 macrophages (M2), highly expressed fibroblast markers (fibroblast associated macrophages, Fibor‐AM), highly expressed SMC markers (SMC associated macrophages, SMC‐AM), and highly expressed redblood cell markers (redblood cells associated macrophages, SMC‐AM) (Figure 4A,C and Table S4). Fibor‐AM (vimentin+/CD68+) and SMC‐AM (ACTA2+/CD68+) were verified by immunofluorescence (Figure S6). The alteration in M1/M2 balance plays a significant role in various diseases or inflammations.^41^, ^42^ An increase of M1 macrophages and a decrease of M2 macrophage was observed in the myometrium of TIL group (Figure 4B, Figure S4A and S4B). Observably, the ST data and staining revealed that M1 (CD86) and M2 (CD163) infiltrated into myometrium, especially in TIL myometrium (Figure 4D,F). Flow cytometry analysis and Western blotting (WB) were performed to confirm the proportion of M1 (CD86) and M2 (CD163) in the myometrium, and the results revealed an increase in M1 macrophage infiltration in the myometrium in the TIL group (Figure 4E,G and Figure S4C). The differential cytokines and their receptors such as CCL3, CCL3L1, CCL4, CCL4L2, and CSF1R were mainly expressed in M2, and down‐regulated in TIL myometrium (Figure 4H,I).

### 2.5 The expression of cytokines in the T and NK cell subpopulations

**FIGURE 5:**
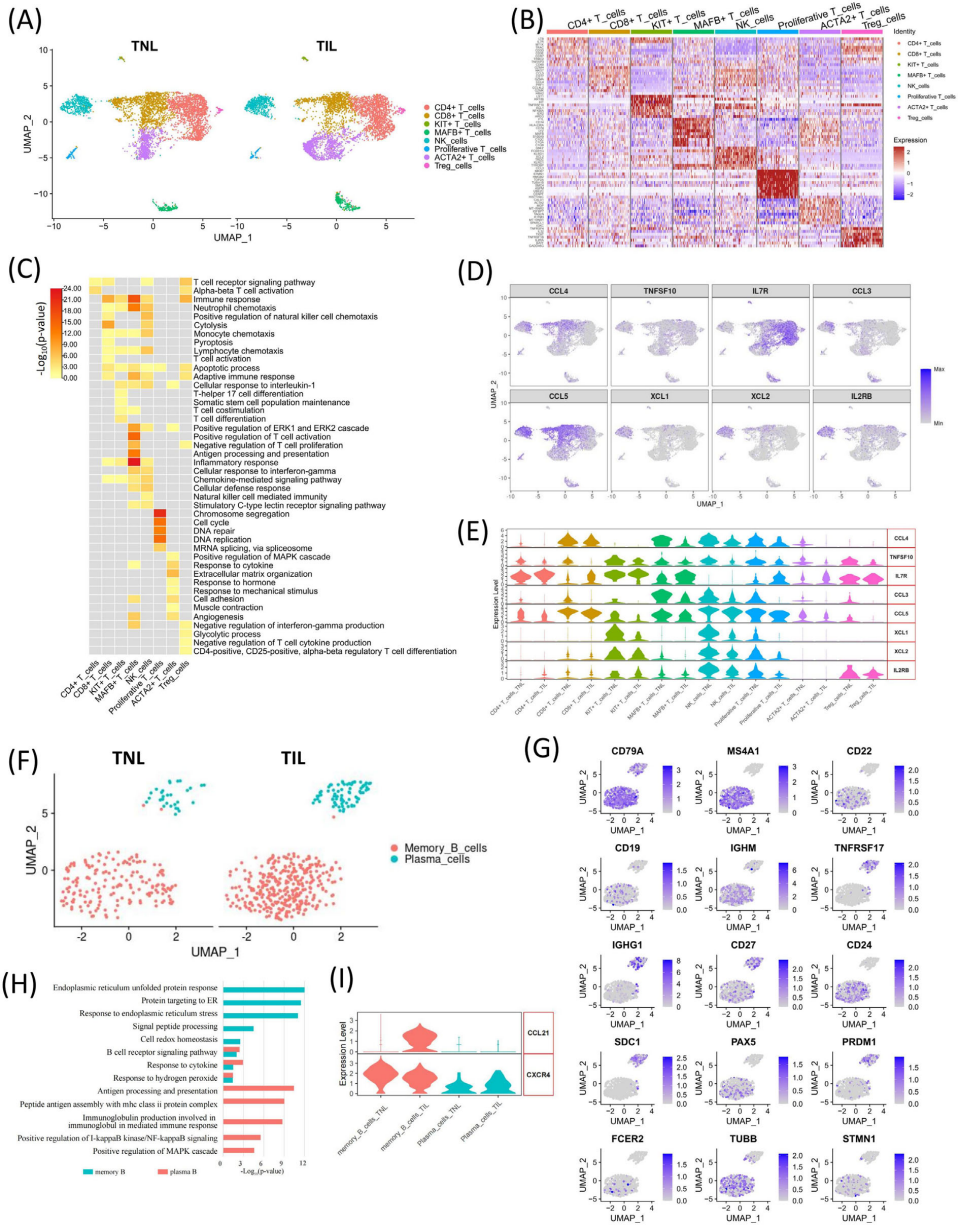
The heterogeneity of T cells, NK cells, and B cells in myometrium during labour. (A) UMAP visualization of T and NK subpopulations divided into groups in scRNA‐seq. (B) Heatmap of the relative expression of the top ten highly expressed genes in each T and NK cell subpopulation in scRNA‐seq (showed 300 cells for each subpopulation). (C) Heatmap of the representative GO terms enriched by marker genes in each T and NK cell population. (D) UMAPs of the expression of the differentially expressed cytokines and receptors in T and NK subpopulations. (E) Violin plot of the expression of differentially expressed cytokines and receptors in each T and NK subpopulation. (F) UMAPs visualization of B cells subpopulations divided into groups. (G) UMAPs of the expression of the marker genes for each B cell subpopulation. (H) GO enrichment analysis of the DEGs in each B cell subpopulation. (I) Violin plot of the differentially expressed cytokines and receptors in each B cells subpopulation.

Immune cells, especially T and NK cells, are considered to play important roles in an adaptive and innate immune response.^25^ To study the changes in the subpopulation of T cells and NK cells, we profiled and analyzed the T cells and NK cells in the myometrium of TNL and TIL groups. In the T cells and NK cell clusters, we identified regulatory T (Treg) cells, CD4+ T cells, CD8+ T, cells KIT+ (CD117) T cells, MAFB+ T cells, NK cells, ACTA2+ T cells (the T cells that highly express SMC markers, SMC‐AT), and proliferative T cells, and proliferative T cells (Figure 5A,B, and Table S4). CD8+ T cells and NK cells produced more chemotaxis than other types of immune cells. MAFB+ T cells are mostly enriched in immune response (Figure 5C), and MAFB promotes T cell growth via the Notch signalling pathway.^43^ The expressions of CCL3, CCL4, CCL5, XCL1, XCL2, TNFSF10 and IL2RB were down‐regulated in TIL myometrium, except IL7R (Figure 5D,E). CCL4 is expressed in CD8+ T cells, NK cells, and proliferative T cells. CCL3 was primarily expressed in MAFB+ T cells and NK cells. CCL5 is expressed in CD4+ T cells, CD8+ T cells, NK cells, and proliferative T cells. TNFSF10 is partially expressed in all T cells and NK cell subpopulations. IL2RB, XCL1, and XCL2 were expressed in KIT+ T cells and NK cells and decreased in labouring myometrium. Studies have demonstrated that XCL 1 and XCL2 contribute to regulating T cell development, which plays an important role in establishing self‐tolerance.^44^, ^45^ An increase in the expression of IL7R was observed in MAFB+ T cells, ACTA2+ T cells, CD4+ T cells, and KIT+ T cells. The ST analysis revealed a few spots of T cell subpopulation markers.

### 2.6 B cell subpopulations in TNL and TIL myometrium

The scRNA‐seq results showed that the proportion of B cells was low (0.50%) in myometrium from TNL and TIL groups There was no significant difference in the proportion of B cell subpopulation between the two groups; therefore, it is likely that B cells may contribute indirectly to immune regulation during delivery. Therefore, we analyzed the B cell subpopulations and the transcriptional changes of inflammation‐related genes. With the marker of B cells (CD79A+), Memory B cells (CD79A+, MS4A1+, SDC1‐) and Plasma cells (CD79A+, SDC1+, PRDM1+ and PAX5‐),^46^ were clustered into memory B cells and plasma cells (effector B cells) (Figure 5F,G). The genes with specific high expression in memory B cells were enriched in endoplasmic reticulum stress. The plasma cells participated in the antigen processing and presentation during immune responses (Figure 5H and Table S5). We also test the expression of the markers of B1, B2 and follicular B cells,^46^ but it did not clustered cells into subgroups in UMAP (Figure 5G). The expression of CCL21 was increased in memory B cells in TIL myometrium, which is chemotactic to activate T cells in vitro.^47^, ^48^ The expression of CXCR4 in B cells was down‐regulated in TIL, compared in which of TNL groups (*p*‐value = 0.0017). The expression of CXCR4 in memory B cells was down‐regulated in TIL group (*p*‐value = 5.99E‐06). And showed no difference for plasma cells (p‐value = 0.0715). (Figure 5I and Figure S3C).

### 2.7 Abundant neutrophils infiltration in the myometrium during labour

**FIGURE 6:**
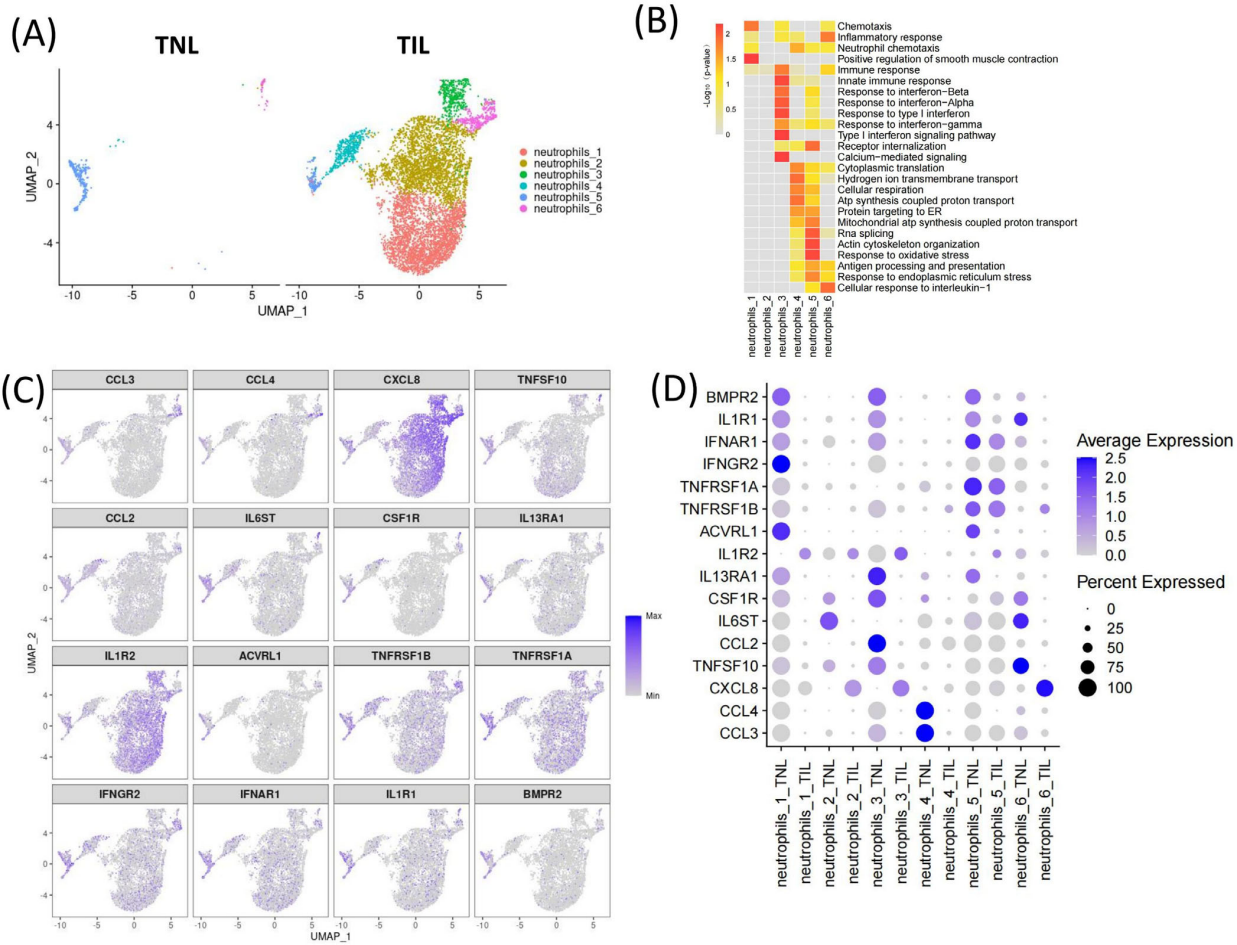
Involvement of neutrophils in the immune response in myometrium during labour. (A) UMAP visualization of neutrophils subpopulations divided into groups. (B) Heatmap of the GO terms enriched by the DEGs in each neutrophil subpopulation. (C) UMAP and (D) Dot plot of the expression of differential expressed cytokines and receptors in each neutrophil subpopulation.

The number of neutrophils in the peripheral blood of TIL group was high compared to the TNL group. It has been reported that the neutrophils highly expressed surface activation markers of CD11a, CD11b and CD62L in TIL, which shows enhanced migration.^49^ In humans and rodents, neutrophils migrate to reproductive tissues during labour,^50^, ^51^ and participate in the labour process by releasing pro‐inflammatory cytokines. Our scRNA‐seq data results reveal an increase in CXCL8 expression in neutrophils in TIL myometrium. Neutrophils were clustered into six subpopulations (Figure 6A), despite the inability of their specific markers to distinguish each subpopulation (Figure 6B and Table S4). The expression of CXCL8 was increased in the neutrophils subpopulations neutrophils‐1, neutrophils‐2, neutrophils‐3, and neutrophils‐6 (Figure 6C). CCL3 and CCL4 were expressed in neutrophils‐4, neutrophils‐5 and neutrophils‐6 subpopulations. IL1B receptors, IL1R1 and IL1R2 showed oppositely different trends. IL1R2 was specifically expressed in neutrophils‐1, neutrophils‐2 and neutrophils‐3 subpopulations and increased in TIL myometrium (Figure 6D). IL1R1 expressed in neutrophils, endothelial, fibroblasts, and trophoblast cells, and decreased in TIL neutrophils (Figure S3D). These results suggested that IL1B—IL1R2 signal was probably more important for the inflammatory response of TIL neutrophils. IL1B—IL1R1 probably played its roles in endothelial, fibroblasts and trophoblast cells during labour (Figure 2E).

### 2.8 Genes associated with contraction in SMC subpopulation

**FIGURE 7:**
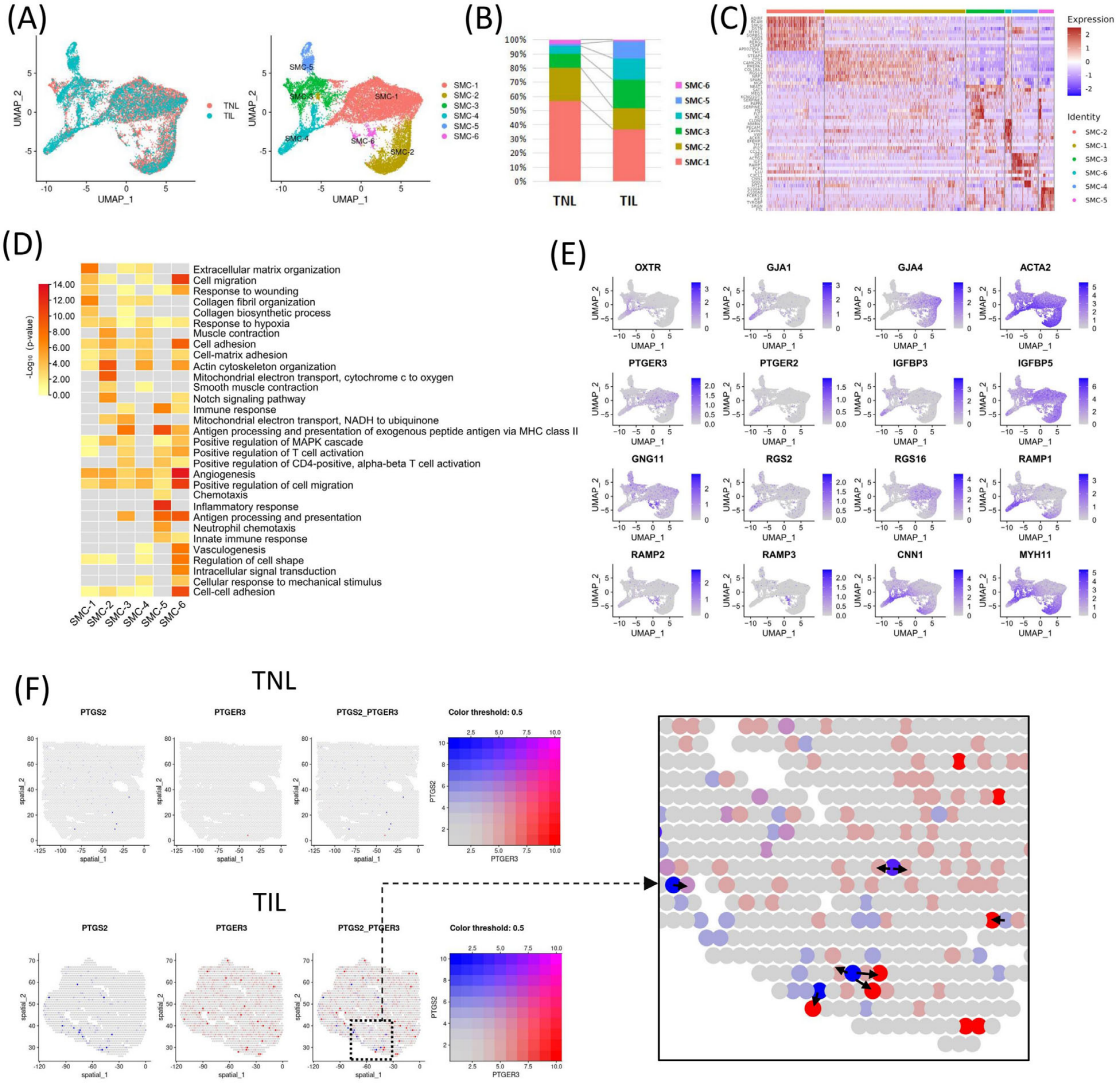
SMC subpopulations and the contraction‐associated genes. (A) UMAP of SMC subpopulations divided into groups. (B) The proportion of SMC subpopulations in the myometrium of the term in non‐labour (TNL) and term in labour (TIL) groups using the scRNA‐seq. (C) Heatmap of the relative expression of the top ten highly expressed genes in each SMC subpopulation. (D) Heatmap of the representative GO terms enriched by marker genes in each SMC cell population. (E) UMAPs of the expression of representative contraction‐associated genes in SMC. (F) Spatial distribution of representative coupled contraction‐associated genes in the myometrium of TNL and TIL groups. Arrows indicate the sources of PTGS2 and its target spots (highly expressed PTGER3).

SMCs are the basic functional unit of the myometrium, which enables the transformation from quiescence to coordinated and intense contractions during labour.^52^, ^53^ The contraction function of SMCs were verified with cell contraction assay (Figure S5F). UMAP clustering of scRNA‐seq results reveals SMC could be characterized into 6 subpopulations (Figure 7A). An increase in SMC‐3, SMC‐4, and SMC‐5 subpopulations was observed in the myometrium from TIL group, while others subpopulation decreased (Figure 7B). The expressions of the top 10 genes with the highest expression level of each subpopulation were shown in the heatmap (Figure 7C). Gene Ontology (GO) analysis revealed that the highly expressed genes of SMC‐3 and SMC‐5 subpopulations were enriched in immune response, and inflammatory response; SMC‐2 and SMC‐4 subpopulations were enriched in muscle contraction; SMC‐1 and SMC‐6 subpopulations were more enriched in cell migration and adhesion biological processes (Figure 7D). Representative contraction‐associated protein genes GJA1 was expressed in partial SMC‐3 and SMC‐4 subpopulations. OXTR was expressed by a small portion of SMC‐1 subpopulation (Figure 7E), consistent with a previous study.^34^ We further studied the representative contraction‐related signal in ST data of myometrium in the TNL and TIL groups. PTGS2 expressed in a part of SMC, monocytes and neutrophils, and its receptors PTGER3 was specifically expressed in SMC‐1 subpopulation (Figure S5A). We could find the some spots that highly expressed PTGS2 or PTGER3 were accessible in the TIL myometrium (Figure 7F).

### 2.9 Inflammation and contraction related crosstalks among each cell populations

**FIGURE 8:**
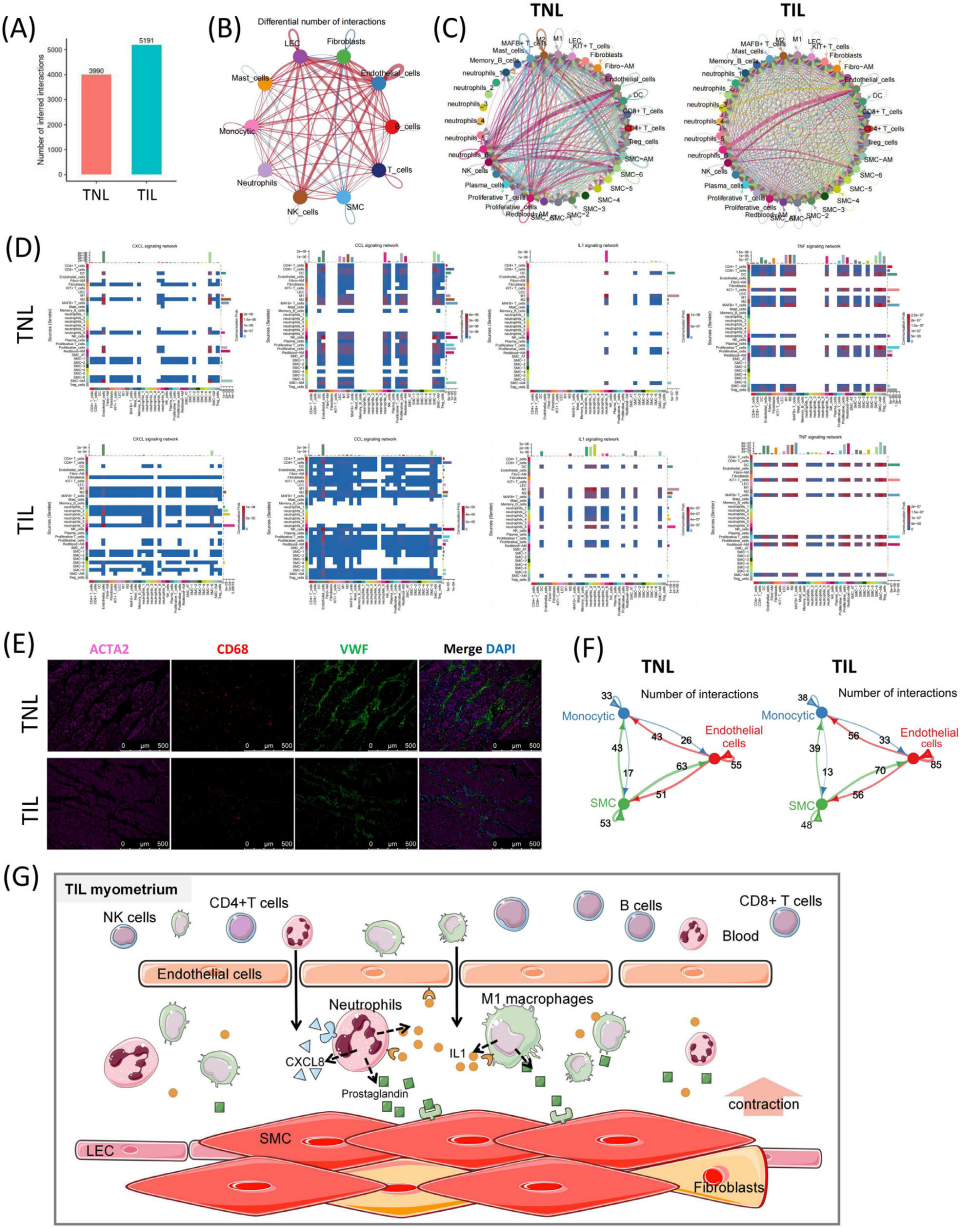
Inflammatory communication in the myometrium of the term in non‐labour (TNL) and term in labour (TIL) groups (A) The total interactions number in the TNL and TIL groups using scRNA‐seq. (B) A differential number of interactions (all ligand‐receptor in CellChatDB database) of major cell populations in the myometrium of TNL and TIL groups. The degree of thickness indicates the interaction number between the sender and the receiver. The red line indicates an increase in the TIL group, and the blue line indicates a decrease in the TIL group. (C) The number of inflammatory interactions (inflammatory ligand‐receptor in CellChatDB database) of cell subpopulations of TNL and TIL groups. The degree of thickness indicates the interaction number between the sender and the receiver. (D) Heatmap of the aggregated cell‐cell communication of representative inflammatory interactions among cell subpopulations in the myometrium of TNL and TIL groups. The colour represents the calculated communication probability. (E) Immunofluorescence indicates the location of three major cell populations in the myometrium of TNL and TIL groups, SMC (ACTA), macrophage (CD68), and endothelial cells (VWF). (F) The number of interactions (all ligand‐receptor in the CellChatDB database) among the three major cell populations in populations myometrium of TNL and TIL groups. (G) The abstract of potential cross‐talks focusing on immune response and SMC contraction in myometrium during labour.

The cell‐cell crosstalks were predicted, and the results revealed increased interactions in the myometrium in the TIL group (Figure 8A). Figure 8B showed that interactions were observed between most major cell populations during labour (Figure 8B). The intercellular communication networks of the myometrium showed that cell–cell communication among endothelial cells, monocytes, LECs, T and NK cells increased in labour. Referring to inflammatory ligand‐receptor in the CellChat DB database (CXCL, CCL, IL1, IL6, TNF, and XCR pathways), endothelial cells, NK cells, SMC‐6, neutrophils‐6, and MAFB+ T cells contribute more interactions in TNL myometrium. The contribution by the interactions between endothelial cells, NK cells, plasma cells, SMC‐6, neutrophils‐6, and neutrophils‐3 subpopulations was high in the myometrium in the TIL group (Figure 8C). The communication probability results indicated that the endothelial cells plays key roles as receivers in CXCL and CCL signaling pathway (Figure 8D). M1 macrophage, DC, and neutrophils‐6 cells sent more IL1 signals during labour. DC, KIT+ T cells, MAFB+ T cells, SMC‐AM, and red blood‐AM cells sent TNF signal with high probability. We further clustered endothelial cells into arterial (IGFBP3) and venous (ACKR1) endothelial cells, capillary (CD36) endothelial cells was too few to find (Figure S5D,E).

To explore the intercellular communication of the uppermost cell populations in TIL and TNL myometrium, we analyzed the cell‐cell communication among the top three most abundant cell populations: SMCs, monocytes, and endothelial cells. The results of immunofluorescence showed the bunched SMC surrounded with endothelial cells, and the macrophages (most abundant monocytic cells) infiltrated the myometrium (Figure 8E). These components formed the main body of the “contractile unit” in the myometrium. The number of cell‐cell communication among SMC, monocytic, and endothelial cells increased in TIL myometrium (Figure 8F and Figure S3B,C). Interestingly, inflammatory interactions were observed between SMCs, monocytes and epithelial cells, with the increased ligand‐receptor pair CXCL8/CXCL3/CCL2_ACKR1 in the TIL group (Figure S5B).

Together, the potential cross‐talks focusing on immune response and SMC contraction in myometrium during labour are abstracted in Figure 8G. During labour, neutrophils and monocytic cells infiltrate into the myometrium and secrete CXCL8 and IL1B to enhance the permeability of endothelial cells and chemotaxis of immune cells. Neutriphils and M1 macrophages expressed PTGS2 to product prostaglandin by the stimulation of CXCL8 and IL1B. Co‐staining with COX‐2 (PTGS2) and the markers of macrophages (CD68) and neutrophilsis (CD66b) were performed to support our hypothesis (Figure S7B). These scRNA‐seq and ST data provide direct evidence for the immune landscape of myometrial cells in TNL and TIL.

## 3 DISCUSSION

Inflammation plays a crucial role in the physiological processes during labour and significantly affects the contractility of the uterus.^25^, ^31^ As a continuation of our previous studies,^11^, ^32^, ^54^ this study provided a comprehensive landscape of changes in myometrium during labour using scRNA‐seq and ST analysis. We have identified monocytes, neutrophils, T cells, NK cells and B cells, and their subpopulations in myometrium. Further, we demonstrated increased levels of monocytes and neutrophils in the myometrium of the TIL group compared with the TNL group. The cytokines and receptors were analyzed to trace their source and target in each immune cell subpopulation. The ST analysis demonstrated the spatial proximity of cytokines and their receptors in myometrium. Next, we discussed the cell‐cell communication among immune cells and SMCs, and determined their role in uterine contractility during labour.

In this study, we focused on the phenomenon of increased myometrial inflammation during labour, and expect to explore the relationship between inflammation and contraction. An increase in the levels of monocytes and neutrophils was observed in the myometrium during labour; however, the levels of other immune cells remained unchanged in the labouring and the non‐labouring myometrium. Further, the balance of M1/M2 macrophages was altered in the myometrium during labour. Previous studies have demonstrated that M1 macrophage polarization occurs at the maternal‐fetal interface and associated with spontaneous term labour.^55^, ^56^ Interestingly, studies have shown that the depletion of macrophages could prevent preterm labour in lipopolysaccharide‐treated mice.^30^, ^57^ Our results demonstrated the changes in the number and phenotype of the macrophage in the labouring myometrium. The macrophage exhibit plasticity and can switch between anti‐ and pro‐inflammatory states, which may be a key factor in myometrial contraction. The previous study did not detect neutrophils in their scRNA‐seq of human myometrium.^34^

IL1B is an important pro‐inflammatory cytokine that activates signal pathways associated with inducing labour, knockout of IL1B in mice could delay labour.^39^, ^58^, ^59^ We identified IL1B was primarily secreted by M1 macrophage. The IL1B receptors like IL1R1, IL1R2, and IL1RAP were widely expressed by neutrophils, and IL1R1 was also expressed by endothelial cells and fibroblasts. The cell number of neutrophil was observed significantly increased in the labouring myometrium, along with increases in IL1R2 expression level, which could be the primary receptor interacting with IL1B. It is noteworthy that the other two pro‐inflammatory cytokines TNF and IL‐6, which are also thought to be produced by macrophages, were not detected in our scRNA‐seq data. However, a significant increase in TNF and IL‐6 protein expression was observed in the labouring myometrium (Figure 1E). In our previous RNA‐seq data of myometrial tissues, the TNF transcripts were also undetectable and the level of IL‐6 mRNA was extremely low (FPKM < 1).^32^ The source of IL‐6 and TNF in myometrium should be studied further.

Studies have shown that neutrophils are present in the myometrium at term, and an increase in the infiltration of neutrophils occurs during labour. Neutrophils stimulate uterine contractions by releasing pro‐inflammatory cytokines and promoting prostaglandins production.^13^, ^60^ CXCL8 is well‐known for its strong neutrophil chemotactic properties.^61^ Our results showed high expression of CXCL8 by the most increased neutrophils during labour, including neutrophils1, 2, 3 and 6 subpopulations. These subpopulations were characterized by biological functions related to inflammatory response, may be the leading cell subpopulations that causing myometrial inflammation. Neutrophil‐5 was the most abundant cell subpopulation in the non‐labouring myometrium, primarily responsible for stress response. Our scRNA‐seq data reveal different functional neutrophil subsets during labour, and it is likely that the synergistic effects of these neutrophil subsets facilitate labour.

SMCs are the primary component of the myometrium and were classified into six subpopulations based on our scRNA‐seq analysis. The results of biological processes enriched by each subpopulation revealed that the marker genes of subpopulations SMC‐1 and SMC‐6 were enriched in cell migration and adhesion. The biological processes enriched by SMC‐2 and SMC‐4 were smooth muscle contraction. The subpopulations SMC‐3 and SMC‐5 play greater roles in mediating immune response. Previous studies on scRNA‐seq of TNL and TIL myometrium reported three subpopulations of SMCs: SMC‐1, which plays a role in smooth muscle contraction; SMC‐2, associated with neutrophil biology; and SMC‐3, associated with the response to IFN‐γ,^34^ that was consistent with our results. A study performed scRNA‐seq on the myometrium with or without leiomyomas classified the SMC into three subpopulations and enriched biological functions like muscle contraction, cell adhesion, response to hormones and energy metabolism.^38^ It should be noted that the sizes of SMC range from 50 to 120 μm in term pregnancy,^34^ although the diameter of suspending SMC is smaller, a proportion of SMCs were with too large size to be captured. Therefore, to minimize SMCs loss, we dissociated tissues with the optimized combination of enzymes as soon as possible. The total SMC accounted for 15.4% of all cells, which was higher than the previous study in which SMCs accounted for 4% of all cells detected.^34^ Single nucleus RNA‐seq would be a good approach to resolve the problems associated with SMCs filtration, but this will lead to loss of cytoplasmic mRNA and failure to capture immune cells.^62^, ^63^

Our study is the first to analyze the spatial pattern of gene expression in both quiescent and contractive states of the myometrium. We explored the spatial orientation of immune cells and their related cytokines as well as receptors. However, the size of SMCs was large, which restricted the display of smaller cells. Some immune cells, like B cells and neutrophils, were hardly detected in the ST data. With the co‐display of cytokines and receptors in ST, we found that the inflammatory signalling interactions were facilitated by their spatial proximity. The ST analysis of labouring myometrium revealed enhanced interaction between CXCL8‐CXCR2, IL1B‐IL1R1, IL1B‐IL1R2, and IL1B‐IL1RAP. the expression of CXCR1 (another receptor of CXCL8) could not be detected in our ST data. Moreover, CCL2‐CCR2 signals were obviously increased in our ST data from labouring myometrium, which consistent with bulk RNA‐seq and cytokine array results, but CCL2‐CCR2 signals could not indicate clearly in scRNA‐seq data. Further, as for the contraction associated genes and associated receptors, the expression of OXTR, PTGS2, and PTGER3 was increased in ST data from labouring myometrium. OXTR and PTGER3 were uniformly distributed in TIL myometrium except for PTGS2. PTGS2 primarily localized in labouring myometrium, and an increase in PTGS2 expression was observed on treatment with antigestagen.^64^ Therefore, combining multiple omics could support each other and reduce technological shortcomings. ST could be used to obtain additional information on the expression and location of genes in the myometrium. More samples will be beneficial to the verification of ST results. Meanwhile, we performed histological staining as a supplement to the validation of scRNA‐seq and ST results. We could only obtain human myometrium in the TNL and TIL group during caesarean sections under the ethical requirements, which is a limitation of this study.

SMCs, immune and endothelial cells, were the main cell types in the myometrium. Inflammatory processes mediate interactions between theses cells, which can enhance myometrial contraction during labour.^31^, ^65^ We believe that the existing “myometrial contraction unit” is necessary and responsible for generating the forceful labour contractions required to deliver the fetus. Mounting evidence have indicated a pivotal correlation between the progesterone and inflammation in labour.^66^, ^67^ Progesterone reduces IL‐1B‐induced PTGS2 expression.^68^, ^69^ Further, M1 macrophage and neutrophils express PTGS2, targeted by IL1B and CXCL8. CXCL8 and IL1B enhance endothelial cell permeability.^70^ This positive feedback leads to inflammatory responses to activate and enhance uterine contraction during labour.

In conclusion, we demonstrated an immune landscape of labouring and non‐labouring myometrium in Chinese women at single‐cell and spatial levels. The changes and interactions between inflammatory factors and immune cell subsets revealed the gene expression pattern involved in pro‐inflammatory processes during labour. The cell‐cell communication analysis demonstrated that monocytes, endothelial cells and SMCs primarily contribute to myometrial contraction. This study helps to elucidate the underlying mechanism of uterine contractions, which might be the ultimate key to managing complicated labour.

## 4 METHODS AND MATERIALS

### 4.1 Sample collection

Myometrial tissues were collected from the lower segment of women who underwent cesarean deliveries at Guangzhou Women and Children's Medical Center (Guangzhou, China). The study included nulliparous women with singleton pregnancies who were at term (≥37 weeks of gestation) and did not have any pregnancy‐associated complications, placenta previa, or receive labour‐augmenting drugs. The samples were divided into two groups: TIL (*n* = 6) and TNL (*n* = 6). Labour was defined as regular contractions occurring less than 3 min apart and cervical dilation of at least 2 cm. The sample collection procedure followed the protocol previously described.^32^, ^54^ The clinical information for the samples was described in Table S1. The myometrial biopsies were immersed with physiological saline and transported to the laboratory for scRNA‐seq, ST, flow cytometry, hematoxylin and eosin staining (H&E), and immunofluorescence. Ethics Committee of Guangzhou Women and Children Medical Center (ethical approval nos. 201915401 and 2018041701) approved this research. All the patients wrote the informed consent.

### 4.2 Dissociation of myometrial tissues for scRNA‐seq

The tissue samples were transferred to a phosphate‐buffered saline (PBS) solution without calcium and magnesium, and any excess tissue was removed. The tissues were cut into 0.5 mm^2^ pieces and then dissociated by a dissociation solution (3 mg/ml collagenase IV, 2 mg/ml papain, and 120 Units/ml DNases I) at 37°C for 20 min. Once the digestion was complete, the resulting cell suspension was passed through a 70‐30 μm stacked cell strainer to remove any debris. The cell was centrifuged and resuspended in 100 μl PBS with bovine serum albumin (BSA). The cells were resuspended into 100 μl using Miltenyi Dead Cell Removal Kit (130‐090‐101, MACS) to remove dead cells. The cell pellet was resuspended in a PBS solution containing 0.04% BSA, and centrifuged at 300 g for 3 min at 4°C. This step was repeated twice. The viability of the cells was assessed using trypan blue. The cell viability should be > 85% for subsequent test. The concentration of suspensions was adjusted to a range of 700–1200 cells/μl after counting by Countess II Automated Cell Counter.

### 4.3 scRNA‐seq library and sequencing

The suspensions of single cells were loaded onto Chromium microfluidic chips to generate single‐cell gel bead emulsions using the Chromium Controller from 10X Genomics, following the manufacturer's instructions. The mRNA from the samples was processed with the Chromium Single‐Cell 3′ kit from 10X Genomics, and full‐length, barcoded cDNA was generated by PCR amplification to produce enough material for library construction. The library were assessed using an Agilent Bioanalyzer 2100, and sequencing was carried out on an Illumina NovaSeq 6000 (LC‐Bio Technology Co.Ltd., Hangzhou, China) using paired‐end multiplexing with a read length of 150 bp.^71^, ^72^

### 4.4 scRNA‐seq data processing

The sequencing data generated were converted into FASTQ format using bcl2fastq software (version 2.20). The CellRanger pipeline (https://www.10xgenomics.com/support/) was applied for sample demultiplexing, barcode processing, and gene counting. The sequencing data were aligned to the human reference genome (Ensembl genome GRCh38). The output from Cell Ranger was then imported into Seurat (version 4.0.2) for dimensional reduction, cell clustering, and generation of figures. All genes expressed < 3 cells were removed. To improve the quality of the data, cells with high variability and low quality were filtered out by removing those with fewer than 200 or more than 6000 genes expressed per cell and more than 25% mitochondrial DNA content. The dimensionality of the remaining cells was then reduced using Seurat to allow for better visualization of the data. UMAP was used to visualize the data. The data were then normalized by “SCTransform” with Seurat. The scRNA‐seq data of all samples were integrated using the “FindIntegrationAnchors” and “IntegrateData” functions in Seurat. To identify clusters within the data, a K‐nearest‐neighbor graph was constructed using “FindNeighbors” function in Seurat. The cells were then grouped together based on the top 30 principal components using “FindClusters” function in Seurat, with a resolution of 1 for the analysis of total scRNA‐seq, a resolution of 0.1 for subpopulation analyses of SMCs and monocytic cells, and a resolution of 0.2 for subpopulation analyses of neutrophils, T cells, B cells and endothelial cells. To identify marker genes for each cluster, the MAST (Model‐based Analysis of Single‐cell Transcriptomics) algorithm was used through the “FindAllMarkers” function in Seurat, with a minimum pct of 0.25.^73^ Differential expression analysis between TNL and TIL groups was performed for each cluster using MAST through the “FindMarkers” function in Seurat, with a minimum pct of 0.5.^74^, ^75^ Pathway enrichment analyses were performed using DAVID (version 6.8) and Gene Set Enrichment Analysis with the Reactome, WikiPathways, and KEGG databases.^76^

### 4.5 Spatial transcriptomics library and sequencing

The tissue samples were harvested, cut into about 10 mm‐thickness blocks, then embedded and frozen in OCT using liquid nitrogen. The tissue blocks were cryosectioned at a thickness of 10 μm and attached to the capture areasbefore proceeding to the next step, followed by H&E staining. The Visium Spatial Gene Expression Slide and Reagent Kit (10 × Visium) were used to process the spatial gene expression data.^77^, ^78^ To prepare the sections for gene expression analysis, they were incubated with permeabilization enzyme for 20 min at 37°C for pre‐permeabilization. After washing the sections with saline sodium citrate, a reverse transcription master mix was added to synthesize cDNA. Following the completion of first‐strand synthesis, 0.08 M KOH was added and incubated for 5 min at room temperature, followed by washing with EB buffer. Second Strand Mix was added to each well for second‐strand synthesis. The Visium spatial libraries were constructed using the Visium Spatial Library Construction Kit from 10 × Genomics. The capture area of the ST is 6.5 × 6.5 mm, with 4992 spots per capture area and a diameter of each spot of 55 μm. The libraries were sequenced using Illumina Novaseq 6000 with a sequencing depth of at least 100,000 reads per spot using paired‐end 150 bp sequencing.

### 4.6 ST data processing

The histology images and FASTQ files were processed using the Space Ranger pipeline (https://support.10xgenomics.com/) and sequence data were aligned to the human reference genome (Ensembl genome GRCh38). The resulting output was imported into Seurat for further analysis, including dimensional reduction, clustering, and data integration. The spots over tissue were only retained for further analysis. A total of 4285 spots were obtained from the myometrium of the TNL group and 1850 spots from the myometrium of the TIL group. The raw counts were normalized using the “SCTransform” function in Seurat. Integrating scRNA‐seq with ST was used the “FindTransferAnchors” function in Seurat according to the previous study.^79^

### 4.7 Cell–cell communication analysis

Expression matrices of scRNA‐seq were processed using the Seurat (version 4.0.2). The cell‐cell communication networks were identified and visualized by CellChat (Version 1.1.2) package following the standard workflow.^32^ Molecule interaction database was used CellChatDB.

### 4.8 Immunofluorescence staining

The myometrium samples were embedded in paraffin and sliced into 5 μm thickness. The tissue sections were then dewaxed and hydrated, followed by antigen retrieval using sodium citrate buffer. The sections were blocked with 10% goat serum for 1 hour, then the sections were stained overnight at 4°C with α‐SMA (ACTA2) (1:300, ab7817, Abcam), VWF (1:500, ab6994, Abcam), tryptase (1:250, 16646‐1‐AP‐100, Proteintech), CD68 (1:250, ab201340‐500, Abcam), vimentin (1:500, ab8978, Abcam), CD3 (1:100, ab135382, Abcam), CD66b (1:100, ab197678, Abcam), COX‐2 (PTGS2) (1:250, ab179800, Abcam), CK‐7 (KRT7) (1:200, ab154334, Abcam), CD86 (1:100, ab269589, Abcam), CD163(1:150, 16646‐a‐AP‐100, Proteintech), followed by Alexa Fluor 647 Goat anti‐mouse secondary antibody (1:500, ab150115, Abcam) or Alexa Fluor 488‐IgG goat anti‐rabbit (1:500, ab150077, Abcam).

Multiple immunofluorescence staining was performed using Goat Anti‐Mouse/Rabbit Multiplex IHC Detection Kit (ZENBIO). After dewaxing, hydration and antigen retrieval, the tissue sections were covered by 3% hydrogen peroxide in the dark for 15 min at room temperature. Then the sections were blocked using 10% goat serum for 1 h and incubated with primary antibodies and secondary antibodies sequentially. The primary antibodies used were as follows: α‐SMA (ACTA2) (1:300, ab7817, Abcam), VWF (1:500, ab6994, Abcam), CD68 (1:250, ab201340‐500, Abcam), vimentin (1:500, ab8978, Abcam). The secondary antibody was Goat anti‐Mouse/Rabbit HRP polymer. Then tissue sections were incubated with TSA 520 Dye (added TSA Enhancer) for 15 min in dark. The steps of antigen retrieval to Dye incubation were repeated until the various tyramine fluorescein substrates are incubated. Finally, the sections were counterstained with the nuclear dye DAPI and visualized using a Leica DMi8 fluorescence microscope.

### 4.9 Immunohistochemical staining

The myometrium samples were embedded in paraffin and sliced into 5 μm thickness. To perform immunohistochemical staining, the Mouse and Rabbit Specific HRP/DAB (ABC) Detection IHC Kit (ab64264, Abcam) was used, following the manufacturer's instructions. Peroxidase quenching was used for the hydrogen peroxidase block. Tissue sections were incubated overnight in a moist chamber at 4°C with primary antibodies CD68 (1:500, ab201340, Abcam), CD3 (1:150, ab135372, Abcam), CD56 (1:20000, ab75813, Abcam), CD19 (1:100, ab134114, Abcam) or CD66 (1:100, ab197678, Abcam). After primary antibody incubation, the tissue sections were incubated with Biotinylated Goat Anti‐Polyvalent secondary antibody for 1 hour. The diaminobenzidine tetrahydrochloride (DAB) chromogen was used for the production of brown colouration. Images were captured by a Leica DMi8 fluorescence microscopy.

### 4.10 Western blotting

The myometrium samples were lysed with RIPA buffer (Beyotime), and the supernatant was collected by centrifugation at 12,000 rpm at 4°C for 5 min. The protein concentration of the supernatant was determined using a BCA assay kit (Thermo Scientific). The proteins were the separated using a SDS polyacrylamide gel electrophoresis (PAGE) and transferred to polyvinylidene fluoride membranes (Millipore). Following blocking with 5% skim milk,, the membranes were incubated overnight at 4°C with primary monoclonal antibodies: rabbit polyclonal β‐actin antibody (1:5000, ab8226, Abcam), CD86 (1:100, ab269589, Abcam), CD163(1:150, 16646‐a‐AP‐100, Proteintech). Then incubated for 1 h with secondary antibodies: HRP‐conjugated Affinipure Goat Anti‐Rabbit IgG (H+L) (1:5000, SA00001, Proteintech) and HRP‐conjugated Affinipure Goat Anti‐Mouse IgG (H+L) (1:5000, SA00001, Proteintech). Proteins were quantified by a ChemiDoc XRS+ system and ImageJ software, β‐actin were used ascontrol.

### 4.11 Flow cytometry

Fresh myometrium samples were dissociated and digested according to the single‐cell preparation procedures. Cells were resuspended with 100 μl fresh stain buffer with 3 μl of CD14 (367115, Biolegend), CD86 (374211, Biolegend) and CD163 (333613, Biolegend). Then stained for 30 min at 4°C, followed by 1 μg of PE goat mouse IgG antibody (405307, Biolegend) for 20 min at 4°C. Flow cytometric analysis was performed on BD LSRFortessa flow cytometer. Data was analyzed by FlowJo software.

### 4.12 Cell contraction assay

SMCs were cultured as per the previous description.^80^ The contractility of SMCs was evaluated by a cell contraction kit (CBA‐201, Cell Biolabs Inc.) according to the manufacturer's instructions. The collagen preparation solution was diluted and mixed with cell suspension, then add to a 24‐well plate for 1 h culture to form solid gel, then add 1 ml medium continue to culture for 48 h. The SMCs were treated with contractile inhibitor (Biacetyl monoxime Diacetyl Monoxime, BPM) (10 μM) to inhibit constriction. After 2 h treatment, the gel was released and measured using a ChemiDoc XRS+ (Bio‐Rad) at 24 h.

### 4.13 Statistical analysis

All statistical analyses and figures were carried out by R (version 4.1.1) and GraphPad Prism software (version 8.0). Bar graphs were displayed as mean ± standard deviation. Comparisons between two groups were using the two‐tailed Student's t‐test, and a *p*‐value < 0.05 was considered statistically significant. The nonparametric Wilcoxon rank sum test in Seurat package was used to analyze the DEGs among subpopulations or groups. *p*‐Value was adjusted by Bonferroni correction.

## CONFLICT OF INTEREST STATEMENT

The authors declare no conflict of interest.

## Supporting information



Supporting Information

Supporting Information

Supporting Information

Supporting Information

Supporting Information

Supporting Information

Supporting Information

Supporting Information
