# Supplementary figures and images for: Integrating single‐cell RNA sequencing with spatial transcriptomics reveals an immune landscape of human myometrium during labour
Source: Clin Transl Med. 2023 Apr 24;13(4):e1234. doi: 10.1002/ctm2.1234 (PMC10126311; doi:10.1002/ctm2.1234)

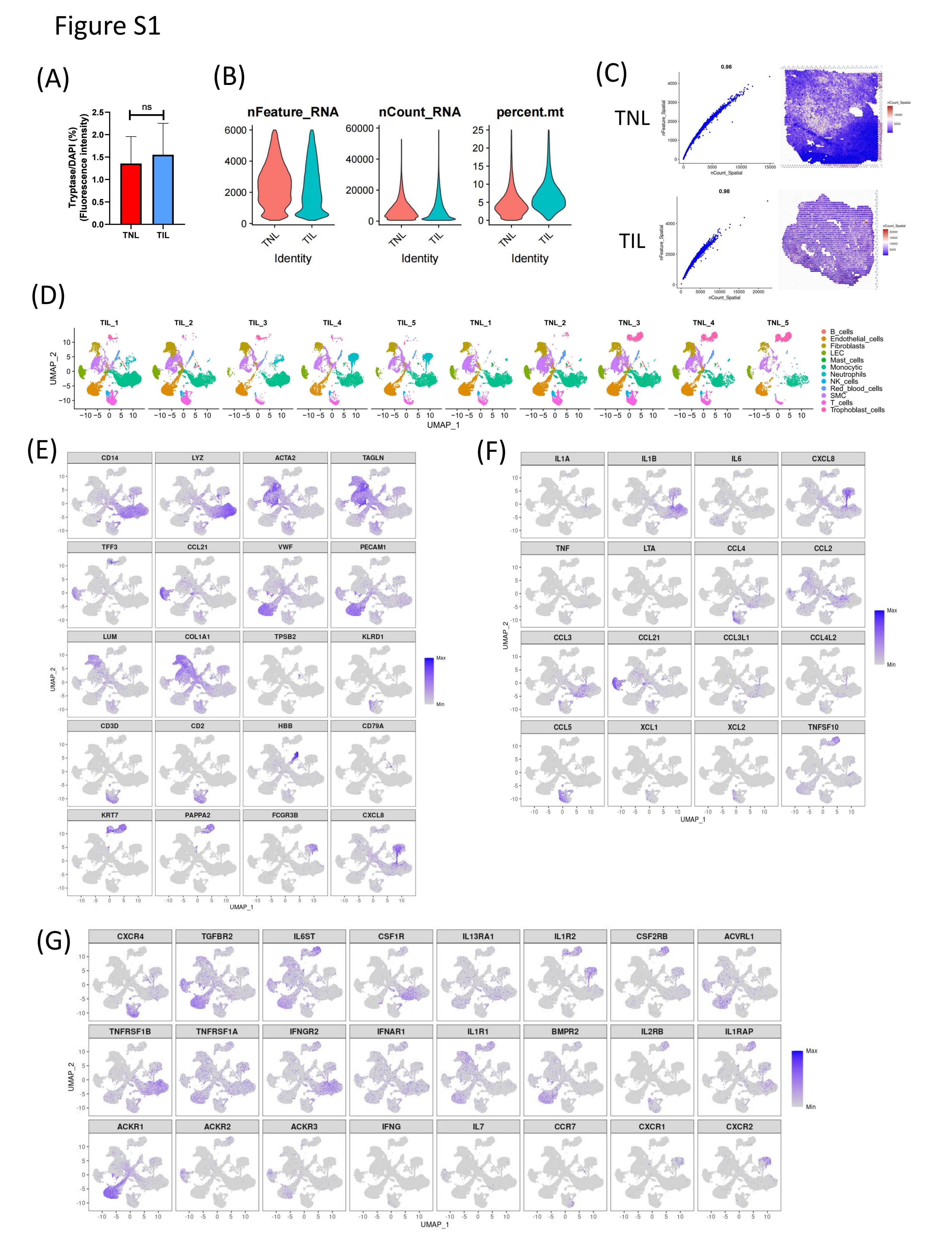

Supplement: Supplementary file 1 — Supporting Information [file CTM2-13-e1234-s006.jpg]

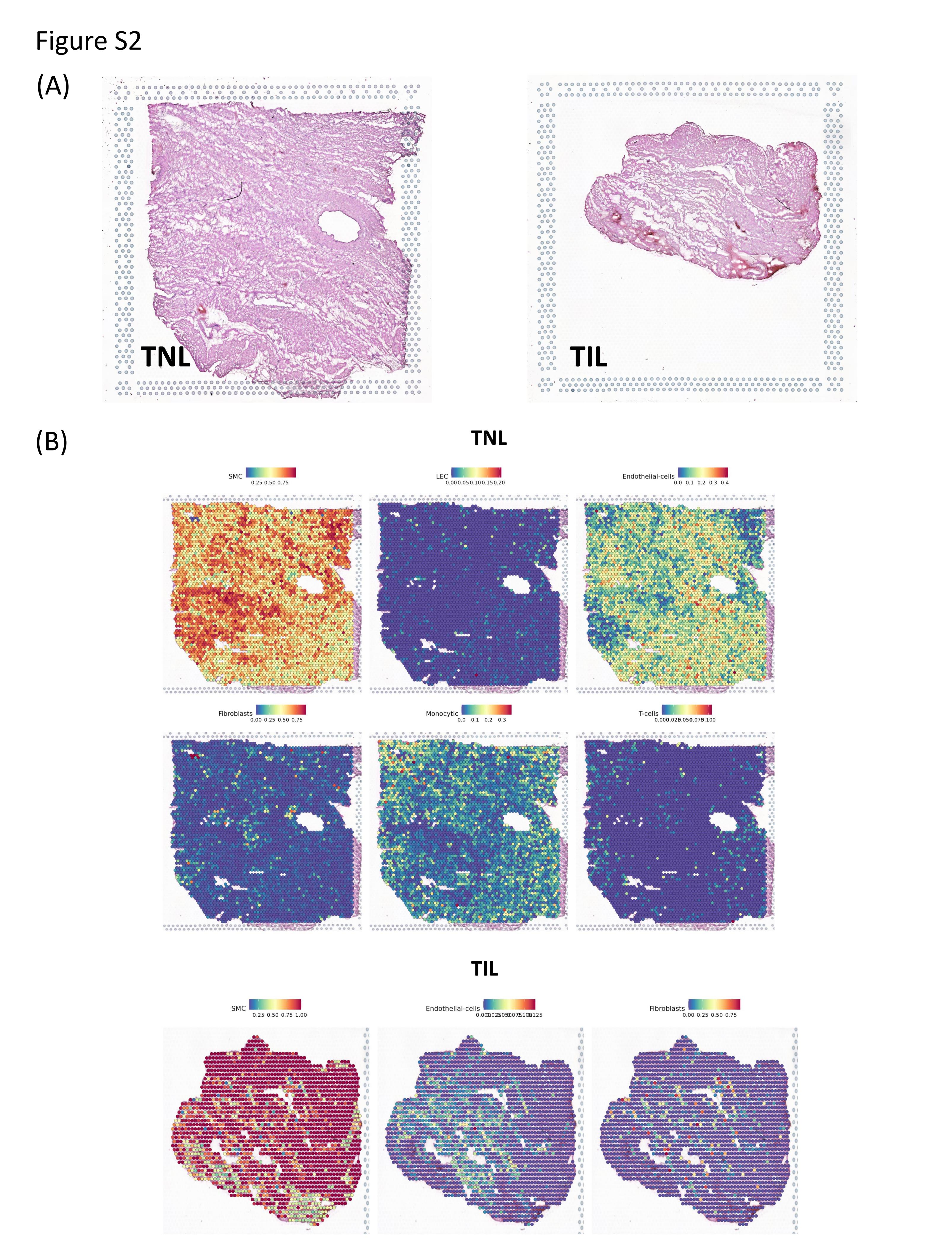

Supplement: Supplementary file 2 — Supporting Information [file CTM2-13-e1234-s007.jpg]

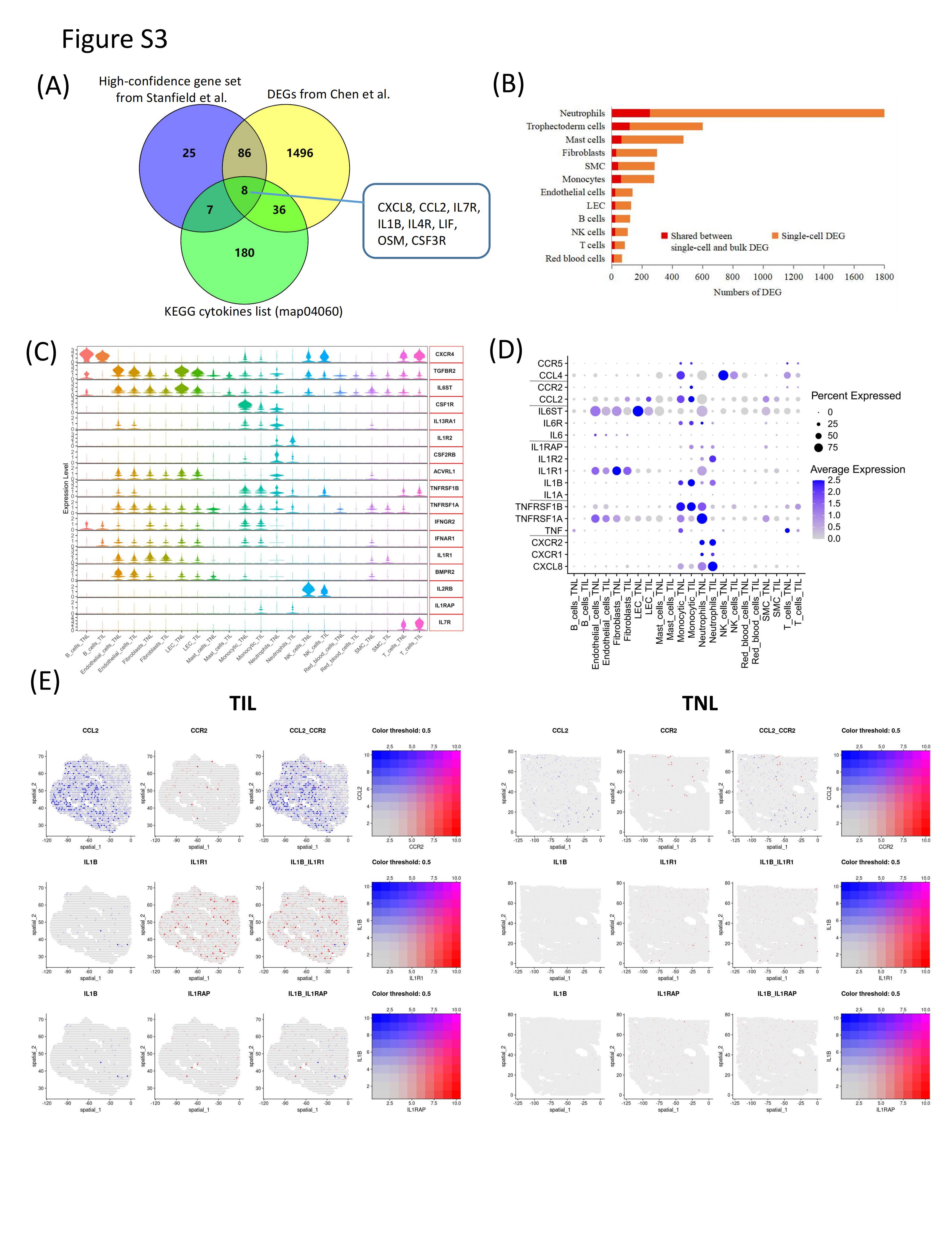

Supplement: Supplementary file 3 — Supporting Information [file CTM2-13-e1234-s008.jpg]

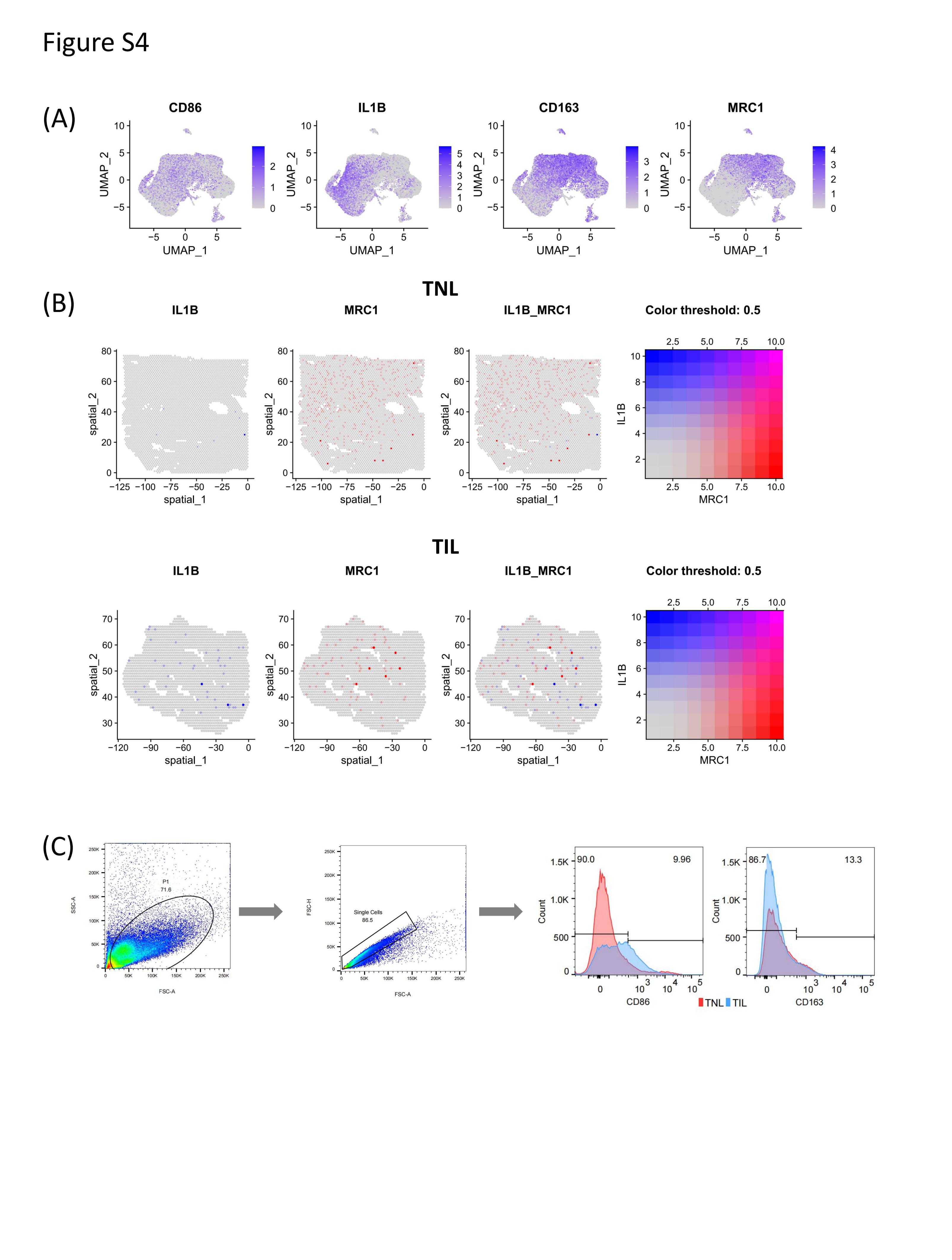

Supplement: Supplementary file 4 — Supporting Information [file CTM2-13-e1234-s002.jpg]

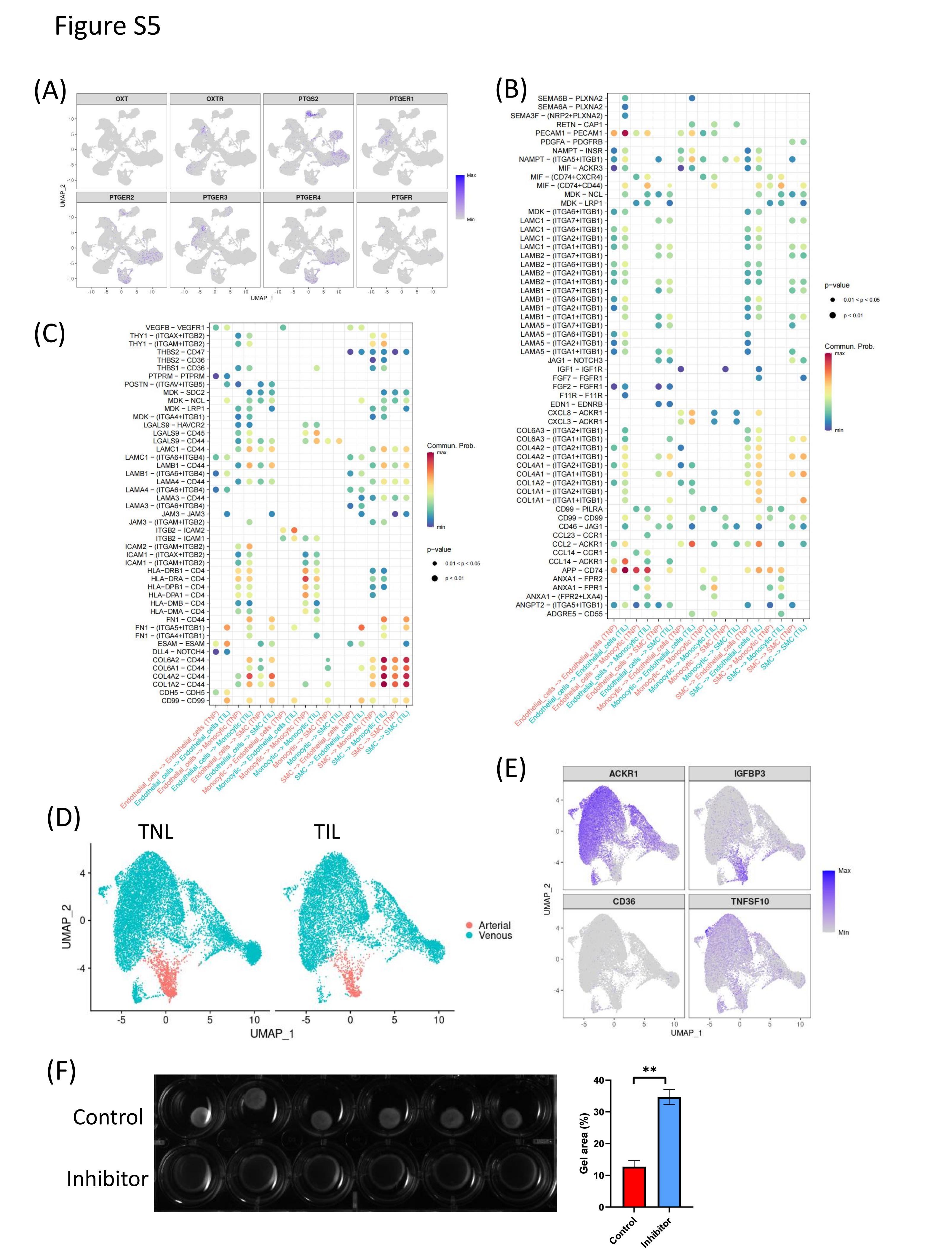

Supplement: Supplementary file 5 — Supporting Information [file CTM2-13-e1234-s003.jpg]

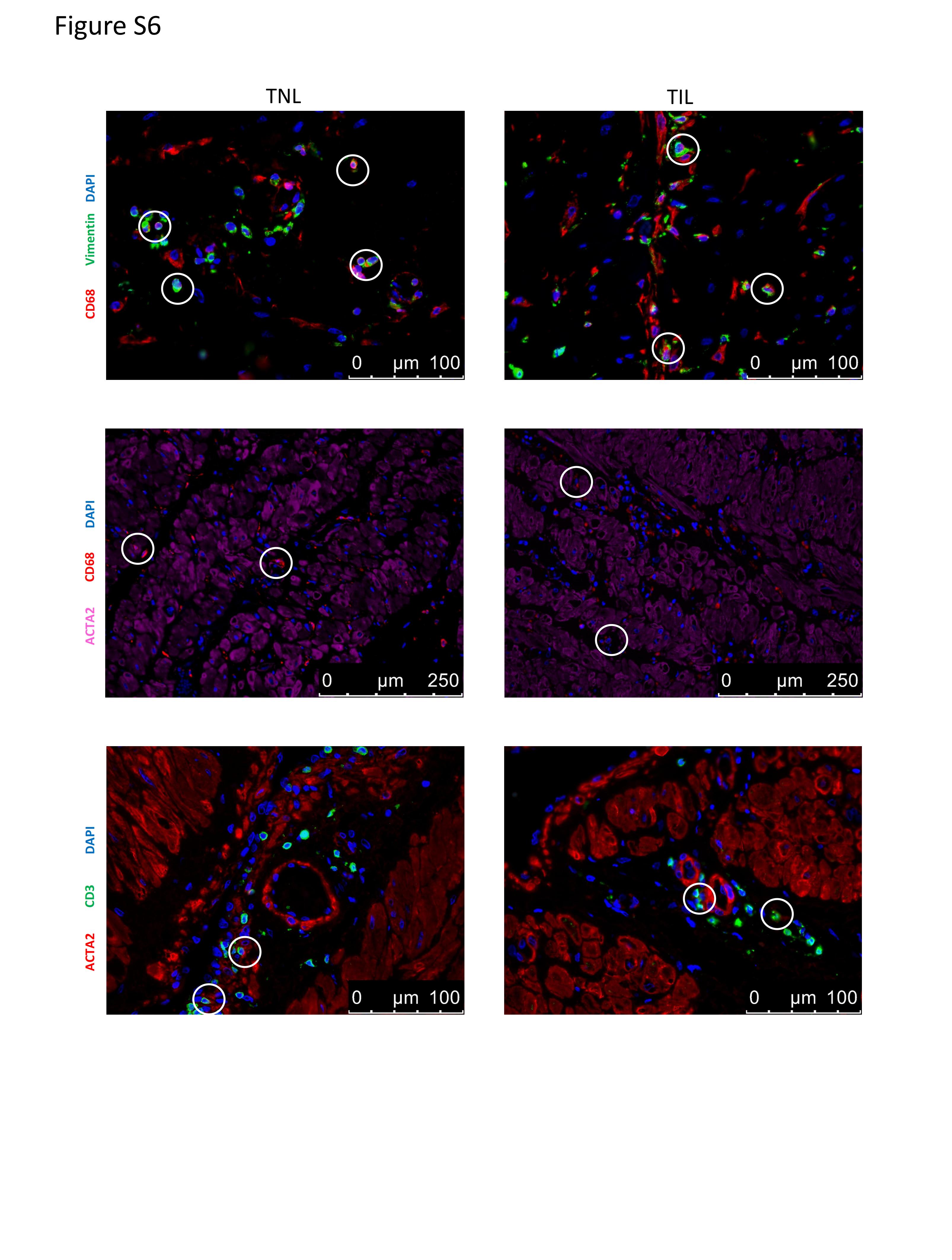

Supplement: Supplementary file 6 — Supporting Information [file CTM2-13-e1234-s004.jpg]

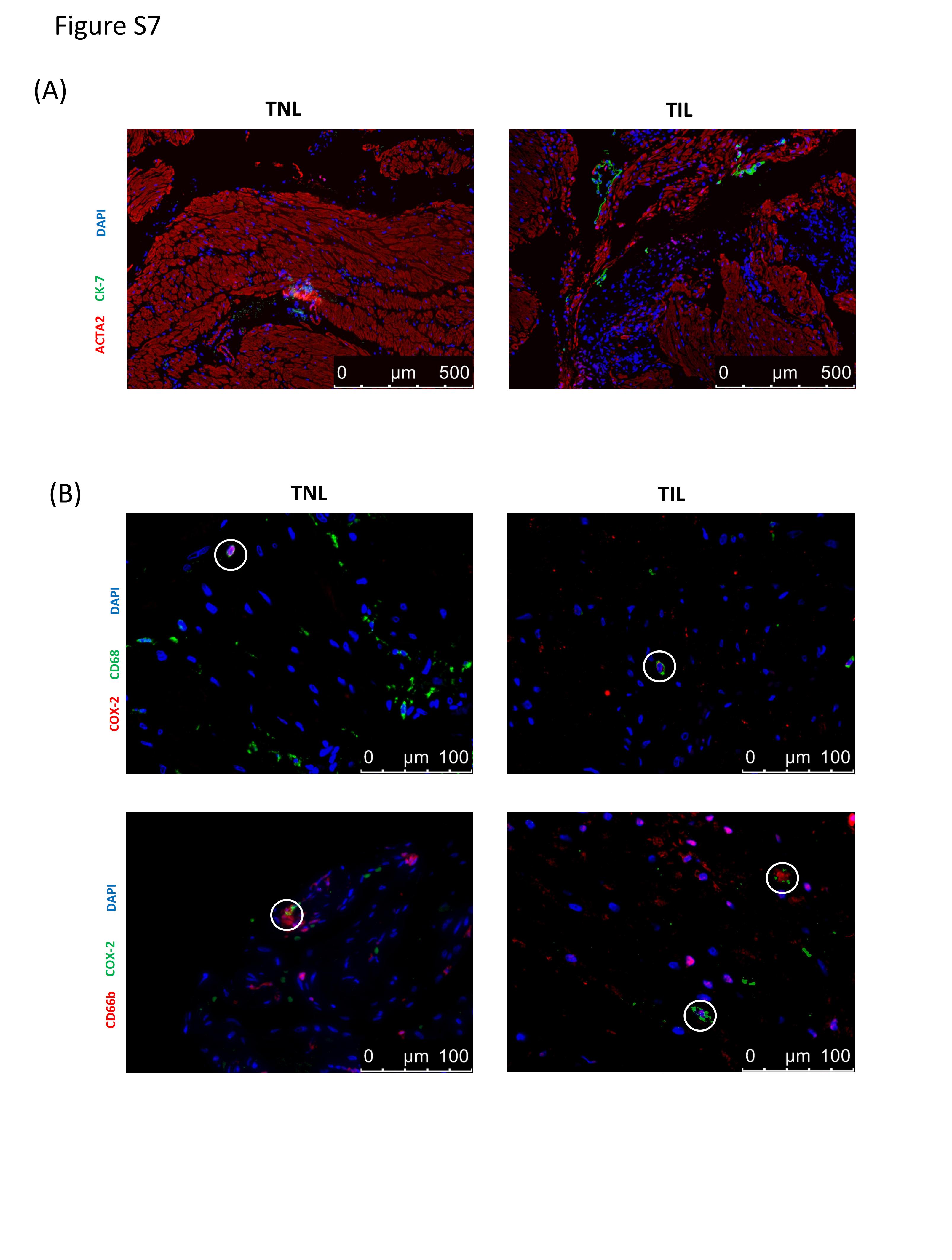

Supplement: Supplementary file 7 — Supporting Information [file CTM2-13-e1234-s001.jpg]
